# Inflammation–neurotrophin synergy of Xiao-yao-san-type botanical drug formulations in depressive disorders: a qualitative synthesis of recent human studies with taxonomic and compositional characterisation

**DOI:** 10.3389/fphar.2026.1718573

**Published:** 2026-03-18

**Authors:** Liu Han, Qun Liang

**Affiliations:** 1 Department of Behavioural Science and Health, University College London, London, United Kingdom; 2 First Affiliated Hospital of Heilongjiang University of Traditional Chinese Medicine, Harbin, China

**Keywords:** depressive disorder, HPA axis regulation, network pharmacology, PI3K-Akt signalling pathway, Xiaoyao formula

## Abstract

**Background:**

Depressive disorders represent a major contributor to the global burden of disease, with persistently rising prevalence rates posing significant challenges to individual quality of life and public health systems. Existing first-line medications such as selective serotonin reuptake inhibitors (SSRIs) and serotonin-norepinephrine reuptake inhibitors (SNRIs) typically require 2–4 weeks to take effect, with complete remission rates below 60%. Approximately one-third of patients discontinue treatment within 90 days due to adverse reactions including gastrointestinal discomfort, weight changes, or sexual dysfunction. Consequently, exploring interventions with faster onset and improved tolerability holds significant clinical importance.

**Methods:**

A systematic search of seven databases—PubMed, Embase, Web of Science, Cochrane CENTRAL, CNKI, AMED, and Scopus—identified randomised controlled trials (RCTs) and mechanism studies published between 2010 and 2025. A qualitative synthesis method analysed clinical efficacy and adverse reactions, integrating evidence from metabolomics, epigenetics, and network pharmacology. Botanical drug identification was performed in accordance with ConPhYMP guidelines, with all species names validated taxonomically against the Medicinal Plant Names Services (MPNS) and Plants of the World Online (POWO) databases.

**Results:**

Twenty-one RCTs (n = 2,766) and three mechanistic studies were included. Findings indicated that Xiaoyao Formula *may* exert earlier effects than SSRIs (descriptive trend: 1–2 weeks vs. 2–4 weeks), with comparable or *preliminary evidence of* superior remission rates and lower residual symptom incidence. Adverse reactions, particularly gastrointestinal discomfort and sleep disturbances, were significantly reduced. No serious adverse events, hepatotoxicity, or clinically significant drug interactions were reported across the evidence base, although systematic adverse event reporting was incomplete in earlier trials. Mechanistic studies *suggest* a hypothetical sequential pathway—‘inflammation precedes neuroplasticity recovery’—involving downregulation of IL-6 and NLRP3 inflammasome, reduced methylation of the BDNF promoter, and activation of the PI3K-Akt pathway. A three-dimensional Q-marker system (based on UPLC-HRMS) comprising baicalin, ferulic acid, and glycyrrhizic acid provides a preliminary framework for quantifying the metabolite-mechanism-efficacy relationship and may serve as a candidate criterion for cross-centre consistency testing.

**Conclusion:**

Existing evidence preliminarily supports potential advantages of Xiaoyao Formula in treating depressive disorders, including possibly earlier onset of action, good tolerability, and potential additional benefits in female subgroups. However, given limitations such as small sample sizes, short intervention durations (6–12 weeks), and predominantly combination therapy rather than monotherapy comparisons, these conclusions should be regarded as suggestive or indicative findings rather than definitive efficacy. Long-term efficacy and generalisability across populations require further validation. Future studies should conduct multicentre, large-sample clinical trials with ≥24-week follow-up, incorporating wearable digital phenotyping technologies to confirm its application value in precision psychiatry.

## 1 Introduction

Depressive disorders constitute a major contributor to the global burden of disease. The World Health Organisation estimates that approximately 332 million individuals worldwide are affected, accounting for 5% of disability-adjusted life years and annual economic losses approaching one trillion US dollars (World Health Organisation, 2023). Current first-line treatments—selective serotonin reuptake inhibitors (SSRIs) and serotonin-norepinephrine reuptake inhibitors (SNRIs)—face three unresolved clinical challenges: Delayed onset of action (requiring 2–4 weeks), limited full remission rates (below 60%, with 30%–40% of patients experiencing residual symptoms), and high discontinuation rates due to gastrointestinal discomfort, weight changes, and sexual dysfunction, with nearly one-third of young adults discontinuing treatment within 90 days (Cipriani et al., 2018; Sansone and Sansone, 2012). These limitations share a common root in the inadequacy of single-target strategies to address depression’s multi-pathway pathogenesis, thereby catalysing the research paradigm of ‘rational multi-target pharmacology’—the deliberate selection or design of therapeutic interventions capable of simultaneously acting upon multiple complementary biological pathways (Anighoro et al., 2014).

In recent years, understanding of depression’s pathophysiology has expanded beyond the classical monoamine hypothesis to encompass multidimensional interactive networks. Clinical studies indicate that approximately one-third of depressed patients exhibit elevated peripheral inflammatory markers. Pro-inflammatory cytokines such as interleukin-6 and tumour necrosis factor-α can cross the blood-brain barrier to influence central neurotransmitter metabolism (Miller and Raison, 2016). This inflammatory state subsequently inhibits the expression and signalling of brain-derived neurotrophic factor (BDNF), diminishing hippocampal neuroplasticity. Concurrently, activation of the hypothalamic-pituitary-adrenal (HPA) axis leads to sustained cortisol elevation, establishing a vicious cycle involving the ‘inflammation-BDNF-stress axis’ (Duman and Monteggia, 2006). The identification of this triadic mechanism provides a clear biological target framework for multi-target intervention strategies.

As a classic formula in traditional Chinese medicine for treating affective disorders, Xiaoyao San’s multi-metabolite, multi-target pharmacological properties make it an ideal candidate for testing the aforementioned multi-target hypothesis. First documented in the Song Dynasty’s Taiping Huimin Heji Ju Fang, this formula comprises eight botanical drugs: Bupleuri Radix, Angelicae Sinensis Radix, Paeoniae Radix Alba, Atractylodis Macrocephalae Rhizoma, Poriae Sclerotium, Mint, Ginger, and Glycyrrhizae Radix et Rhizoma (honey-processed). Its traditional efficacy lies in soothing the liver and resolving depression, fortifying the spleen, and nourishing blood. Modern pharmacological research indicates that saikosaponins from Bupleuri Radix possess anti-inflammatory and hypothalamic-pituitary-adrenal axis regulatory activity. Bioactive metabolites in Angelica and Paeonia alba regulate monoamine neurotransmitter levels, while Poriae Sclerotium polysaccharides exhibit neuroprotective effects (Zhang et al., 2022; see Table A for complete taxonomic characterisation). This multi-metabolite synergistic effect, formed through the ‘sovereign-minister-assistant-messenger’ formula, theoretically aligns closely with the multi-target intervention requirements of the ‘inflammation-neurotrophic factor-stress axis’ triad. Derived formulations, including Jiawei Xiaoyao Wan (enhanced with Gardeniae Fructus (Gardenia jasminoides J. Ellis [Rubiaceae]) and Moutan Cortex (Paeonia × suffruticosa Andrews [Paeoniaceae]) to strengthen heat-clearing and blood-activating effects) and Danshi Xiaoyao San, are widely employed in China and East Asia as adjunctive therapies for depression. Previous systematic reviews have examined modified Xiaoyao powder for specific populations such as postpartum depression (Hu et al., 2024), yet none has integrated clinical evidence with multi-target mechanistic data.

However, advancing Xiaoyao-type formulas from empirical use to evidence-based recommendations faces three critical obstacles: clinical trials predominantly employ combination therapy designs, making it difficult to isolate the independent contribution of Xiaoyao formulas from synergistic effects; mechanism studies remain fragmented across network pharmacology predictions, metabolomics associations, and epigenetic observations, lacking integration into testable pathway models; and the lack of standardised quality control systems for batch-to-batch variability in TCM multi-botanical drug formulations hinders the comparability of multicentre studies and international translation. To bridge these gaps, this review systematically retrieved Chinese and English studies published between 2010 and 2025, aiming to address two core questions: What is the strength of clinical evidence for Xiaoyao formulas in alleviating depressive symptoms? Can existing mechanistic data support the multi-target synergistic hypothesis of ‘inflammation preceding neuroplasticity recovery’?

**FIGURE 1:**
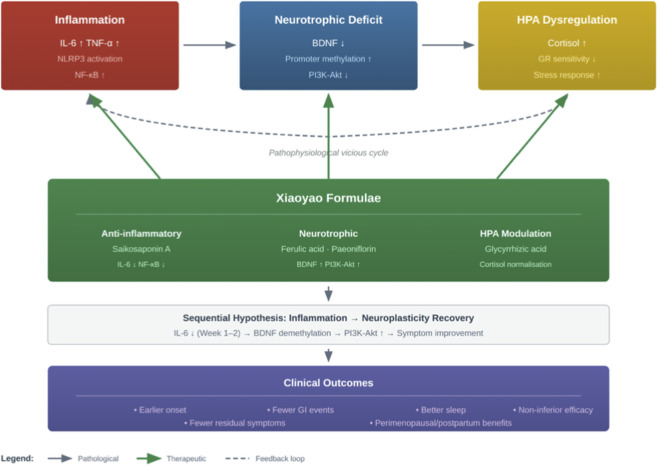
Hypothesised inflammation-neurotrophic factor synergistic mechanism of Xiaoyao formulae in depression.

This review contributes academically on three levels. Regarding evidence integration, it presents the first stratified evaluation of randomised controlled trials on Xiaoyao-type formulas for depressive disorders, distinguishing between macro-level effect sizes and the mechanistic exploration value of individual trials. Theoretical construction: A working hypothesis of the ‘inflammation-neurotrophic factor synergistic mechanism’ is proposed, providing a unified interpretative framework for disparate multi-omics findings. Methodology: Building upon existing analytical chemistry and biomarker research, a three-dimensional Q-marker conceptual framework is introduced, offering an operational research roadmap for quantifying the ‘metabolite-mechanism-efficacy’ relationships within traditional Chinese medicinal formulae. Figure 1 depicts the hypothesised “inflammation precedes neuroplasticity recovery” pathway underlying Xiaoyao formulae’s antidepressant effects.

## 2 Research methodology

### 2.1 Data sources and retrieval strategy

A comprehensive literature search was conducted across seven electronic databases to identify studies examining the efficacy of Xiaoyao San/Pills in treating depression. Primary clinical evidence databases included PubMed (via NCBI), Embase (via Ovid), Web of Science Core Collection (via Clarivate), Cochrane CENTRAL (via Cochrane Library), and CNKI (via CNKI Scholar). For studies on complementary mechanisms and grey literature, AMED (via EBSCOhost) and Scopus (via Elsevier) were additionally searched.

The search period spanned from 1 January 2010 to 30 January 2026, with no language restrictions, though Chinese and English literature were prioritised. The final search date was 30 January 2026.

The search strategy combined intervention terms with disease terms, incorporating mechanism terms where appropriate. It utilised controlled vocabularies (MeSH, Emtree, CNKI subject headings) and free-text terms specific to each database’s indexing system.

Intervention terms included: “Xiaoyao San” OR “XiaoYaoSan” OR “Xiao Yao San” OR “Xiaoyao Wan” OR “XYS” OR “Danzhi Xiaoyao”.

Disease terms include: depress* OR “major depressive disorder” OR “depression”.

PubMed Search Example: ((“Xiaoyao San” [MeSH] OR “XiaoYaoSan” [Title/Abstract] OR “Xiao Yao San” [Title/Abstract] OR “Xiaoyao Wan” [Title/Abstract] OR “XYS” [Title/Abstract] OR “逍遥丸” [Title/Abstract] OR “逍遥散” [Title/Abstract]) AND (depress [Title/Abstract] OR “major depressive disorder”)).

CNKI Search Syntax Notes: To ensure compliance with Cochrane Systematic Review methodological standards, the following revised CNKI search strategy addresses intervention heterogeneity (excluding irrelevant formulas) and insufficient search sensitivity (expanding to all fields).

Please utilise this within CNKI’s Advanced Search interface, setting the search field to “Topic”. When employing Command Search mode, use the field codes TKA(Title, Keywords, Abstract) or SU(Topic, subject to CNKI version definitions; Topic covering all fields is recommended).

Recommended search formula (copy the following): (Topic = Xiaoyao Powder OR Topic = Xiaoyao Pills OR Topic = Jiawei Xiaoyao OR Topic = Dan Zhi Xiaoyao) AND (Topic = Depression OR Topic = Depressive Syndrome OR Topic = Affective Disorder OR Topic = Anxiety-Depression).

#### 2.1.1 Botanical drug identification and compositional characterisation

In accordance with the ConPhYMP (Consolidated Standards for Reporting Phytopharmacological and Multi-component Pharmacological Research) guidelines (Heinrich et al., 2022) and the Society for Medicinal Plant and Natural Product Research’s (GA) (2025) best-practice recommendations, the following section systematically characterises the botanical drug formulations evaluated in the included studies. All species names have been validated taxonomically against the Medicinal Plant Names Services (MPNS) portal and Plants of the World Online (POWO), following the nomenclatural conventions recommended by Rivera et al. (2014). Drug names are assigned according to the Chinese Pharmacopoeia (Chinese Pharmacopoeia Commission, 2020).

Xiaoyao formulas (Xiaoyao wan, Free and Easy Wanderer Plus, Xiaoyao pill or Xiaoyao san), first recorded in the Song dynasty text Taiping Huimin Heji Ju Fang (1078–1085 CE), is a traditional Chinese botanical drug formulation comprising eight botanical drugs in a defined weight ratio (5:5:5:5:5:4:1:5). The base formula and its principal variants, which were evaluated in the included studies, are characterised below. The composition of each variant was verified against the original publications of the included trials and cross-referenced with the monographs in the Chinese Pharmacopoeia (Chinese Pharmacopoeia Commission, 2020). Where original studies did not provide complete compositional data, this is explicitly noted in Table 1.

**TABLE 1:** Taxonomic identification and pharmacopeial characterisation of botanical drugs in Xiaoyao-type formulations.

Botanical drug (Chinese name)	Validated species name [family; pharmacopeial drug name]	Part used	Weight ratio	Formulation
Chaihu (柴胡)	Bupleurum chinense DC. or B. scorzonerifolium Willd. [Apiaceae; Bupleuri Radix]	Dried root	5	Base formula
Danggui (当归)	Angelica sinensis (Oliv.) Diels [Apiaceae; Angelicae Sinensis Radix]	Dried root	5	Base formula
Baishao (白芍)	Paeonia lactiflora Pall. [Paeoniaceae; Paeoniae Radix Alba]	Dried root (peeled)	5	Base formula
Baizhu (白术)	Atractylodes macrocephala Koidz. [Asteraceae; Atractylodis Macrocephalae Rhizoma]	Dried rhizome	5	Base formula
Fuling (茨苓)	Wolfiporia cocos (F.A. Wolf) Ryvarden and Gilb. [Polyporaceae; Poriae Sclerotium]	Dried sclerotium	5	Base formula
Gancao (甘草)	Glycyrrhiza uralensis Fisch. ex DC. or *G. glabra* L. [Fabaceae; Glycyrrhizae Radix et Rhizoma]	Dried root and rhizome (honey-processed)	4	Base formula
Bohe (薄荷)	Mentha haplocalyx Briq. [Lamiaceae; Menthae Haplocalycis Herba]	Dried aerial parts	1	Base formula
Shengjiang (生姜)	Zingiber officinale Roscoe [Zingiberaceae; Zingiberis Rhizoma Recens]	Fresh rhizome	5	Base formula
Zhizi (栀子)	Gardenia jasminoides J. Ellis [Rubiaceae; Gardeniae Fructus]	Dried ripe fruit	Added	Danzhi/Jiawei variant
Mudanpi (牡丹皮)	Paeonia × suffruticosa Andrews [Paeoniaceae; Moutan Cortex]	Dried root bark	Added	Danzhi/Jiawei variant

Weight ratios refer to the standard Xiaoyao Wan formulation as specified in the Chinese Pharmacopoeia (Chinese Pharmacopoeia Commission, 2020). The Danzhi Xiaoyao (also termed Jiawei Xiaoyao) variant adds Gardeniae Fructus and Moutan Cortex to the base formula to enhance heat-clearing and blood-cooling properties. Wolfiporia cocos is a fungal organism, not a plant species, but is included here as an integral part of the formulation as listed in the pharmacopeial monograph. Across the 21 included trials, four distinct preparation types were employed: Xiaoyao Wan/San (standard base formula), Danzhi Xiaoyao San/Wan (base plus Gardeniae Fructus and Moutan Cortex), Jiawei Xiaoyao capsules (encapsulated Danzhi variant), and Free and Easy Wanderer Plus (FEWP; the English designation for Jiawei Xiaoyao San). All species names follow the Chinese Pharmacopoeia (2020 edition) and have been validated against Plants of the World Online (POWO, 2024; https://powo.science.kew.org/) and the Medicinal Plant Names Services (MPNS, 2024; https://mpns.science.kew.org/). The hybrid symbol (×) for Paeonia × suffruticosa Andrews follows POWO nomenclature. Mentha haplocalyx Briq. is the name designated in the Chinese Pharmacopoeia; POWO treats this taxon as a synonym of M. canadensis L. wolfiporia cocos nomenclature follows the chinese pharmacopoeia and index fungorum; recent molecular phylogenetic evidence suggests the East Asian cultivated form may warrant reclassification as Pachyma hoelen (Wu et al., 2020, Front. Microbiol., 11:590788), but the pharmacopeial name is retained here for consistency with clinical trial reporting.

The pharmacological equivalence of these variants has not been established by the three-dimensional Q-marker framework discussed in Section 4.5, and this compositional heterogeneity constitutes a significant limitation of cross-study comparisons within this review.

#### 2.1.2 Component–pathway concordance analysis

The compositional and pharmaceutical differences documented in Section 2.1.1 and Table 1 — particularly the systematic distinction between the six-botanical-drug classical formula and the eight-botanical-drug Danzhi/Jiawei variants incorporating Gardeniae Fructus (geniposide) and Moutan Cortex (paeonol) — create a quasi-experimental structure within the evidence base that this review uses to make mechanistic inferences. As the pharmacological targets of these variable botanical drugs are characterised in independent preclinical literature—geniposide modulates MAPK/PI3K-Akt signalling and inhibits NLRP3 inflammasome assembly, while paeonol suppresses NF-κB-dependent transcription of pro-inflammatory cytokines—the formulation variation across the 21 included trials generates specific, directional predictions about which biomarker outcomes should differ between formulation subgroups and which should remain invariant. Rather than treating this heterogeneity as a confounding factor requiring statistical adjustment or subgroup exclusion, the present review formalises it as a component–pathway concordance analysis, which is a set of *a priori* pharmacological predictions that function as internal validity tests for the mechanistic model developed in the discussion section. This approach transforms what systematic review methodology typically considers a limitation—inconsistent intervention composition—into the primary analytical tool for evaluating mechanistic plausibility.

Three concordance predictions were specified prior to the interpretive synthesis and are evaluated against the available biomarker data in Sections 4.1, 4.2. This difference in magnitude is due to the additional anti-inflammatory effect of these two additional botanical drugs. Secondly, as the HPA axis-modulating properties of Xiaoyao-type formulations primarily derive from Saik saponins in Bupleuri Radix, a botanical drug present in all four variants, cortisol and ACTH normalisation should be comparable across formulation subgroups. This serves as a specificity control, confirming that any observed inflammatory differences reflect the variable botanical drugs, rather than the invariable ones. Thirdly, the sequential mechanistic hypothesis developed in Section 4.1 — that anti-inflammatory action precedes and enables epigenetic normalisation, which in turn permits neurotrophic recovery—generates a transitive prediction. If Jiawei/Danzhi formulations produce stronger upstream inflammatory suppression, they should yield larger downstream effects on DNMT1 expression and BDNF concentration through amplified propagation along the same sequential pathway rather than through a pharmacologically independent mechanism. Any discordance with these predictions—equivalent inflammatory effects across formulation subgroups or enhanced BDNF responses without corresponding inflammatory differences—would constitute evidence against the proposed sequential model, redirecting mechanistic interpretation towards parallel, independent pathway engagement.

An analogous concordance logic extends to the population dimension. The three clinical subgroups identified during screening—primary depressive disorder; depression with somatic comorbidity, such as functional dyspepsia, chronic hepatitis, post-stroke and post-Covid-19; and female reproductive–associated mood disorders, such as perimenopausal depression and premenstrual syndrome—present distinct baseline inflammatory and neuroendocrine profiles that generate differential predictions for pathway engagement. Somatic comorbidity populations, characterised by elevated systemic inflammation secondary to their primary medical condition, are predicted to show larger absolute reductions in inflammatory biomarkers and correspondingly amplified downstream neurotrophic responses if the sequential model is correct. In contrast, primary psychiatric depression patients with lower baseline peripheral inflammation should exhibit relatively greater HPA axis and direct monoaminergic effects. The subtype-stratified qualitative synthesis in Section 4.2 explicitly tests these population-level predictions, and concordance or discordance between the predicted and observed patterns across the three subgroups provides independent validation of the integrative mechanistic hypothesis. Together, the component–pathway concordance analysis (formulation dimension) and the population–mechanism concordance analysis (patient dimension) forms a dual-axis internal validation architecture. This architecture constrains the degrees of freedom of interpretation by requiring the mechanistic model to account for both compositional pharmacology and population pathophysiology. This methodological requirement is absent from previous Xiaoyao formula reviews and, more broadly, from the systematic review methodology for multi-metabolite pharmacological interventions.

### 2.2 Inclusion and exclusion criteria

Inclusion Criteria: 1. Randomised controlled clinical trials evaluating the therapeutic efficacy of Xiaoyao formula in patients with depression; 2. Population-based studies covering large cohorts to assess the efficacy and safety of Xiaoyao formula across diverse populations; 3. Molecular mechanism studies exploring the multi-target effects of Xiaoyao powder in neurotransmitter regulation, HPA axis, and inflammatory pathways.

Exclusion Criteria: Retrospective studies, animal experiments, purely *in vitro* mechanism studies, conference abstracts, and literature where full-text access was unavailable.

### 2.3 Definitions of terms

To ensure conceptual clarity and facilitate cross-cultural understanding of traditional Chinese medicine terminology, this systematic review provides operational definitions for the following key terms.

Xiaoyao formulas (Xiaoyao wan, Free and Easy Wanderer Plus, Xiaoyao pill or Xiaoyao san) refer to a class of classical Chinese medicinal prescriptions derived from or structurally related to Xiaoyao powder. First recorded in the Song dynasty’s *Taiping Huimin Heji Ju Fang* (1078–1085), Xiaoyao powder comprises eight core botanical drugs: Bupleuri Radix (Bupleurum chinense DC. [Apiaceae]), Angelicae Sinensis Radix (Angelica sinensis (Oliv.) Diels [Apiaceae]), Paeoniae Radix Alba (Paeonia lactiflora Pall. [Paeoniaceae]), Atractylodis Macrocephalae Rhizoma (Atractylodes macrocephala Koidz. [Asteraceae]), Poriae Sclerotium (Wolfiporia cocos (F.A. Wolf) Ryvarden and Gilb. [Polyporaceae]), Glycyrrhizae Radix et Rhizoma (Glycyrrhiza uralensis Fisch. ex DC. [Fabaceae]), Menthae Haplocalycis Herba (Mentha haplocalyx Briq. [Lamiaceae]), and Zingiberis Rhizoma Recens (Zingiber officinale Roscoe [Zingiberaceae]). Within this review, Xiaoyao formulas encompass the original Xiaoyao Powder, its classical modified versions (Dan Zhi Xiaoyao Powder, Jiawei Xiaoyao Powder), and modern standardised preparations.

Dan Zhi Xiaoyao Powder (also known as Jiawei Xiaoyao Powder) is a classic modified formula. It incorporates Moutan Cortex and Gardeniae Fructus to the original formula and is traditionally used for conditions where prolonged qi stagnation transforms into heat, corresponding to the TCM syndrome of ‘Liver Qi Stagnation Transforming into Fire’.

Chaihu Shugan Powder is recorded in the Complete Works of Jingyue (1624) and belongs to the category of Chaihu-based antidepressant formulas. This formula shares the primary botanical drug Chaihu (Bupleurum) with Xiaoyao San but emphasises qi regulation and blood activation through the combination of Xiangfu (Cyperus), Chuanxiong (Ligusticum), Zhike (Fructus Aurantii Immaturus), and others.

Liver-Soothing and Depression-Relieving Capsules are a modern standardised Chinese botanical drug preparation approved by the National Medical Products Administration for the treatment of mild to moderate depression. Containing standardised extracts of *Hypericum perforatum* and Acanthopanax senticosus, they embody a contemporary interpretation of the therapeutic principle of ‘soothing the liver and relieving depression’.

The Hamilton Depression Rating Scale (HAMD) is a clinician-administered assessment developed by Hamilton (1960) to quantify depression severity. Studies included in this review employed either the 17-item version (HAMD-17) or 24-item version (HAMD-24). Therapeutic response was typically defined as a ≥50% reduction from baseline, while clinical remission was defined as a final score ≤7 (HAMD-17) or ≤8 (HAMD-24).

Traditional Chinese Medicine Syndrome Rating Scales refer to validated instruments for quantifying TCM-specific symptom syndromes, enabling assessment of therapeutic efficacy at the syndrome level beyond conventional psychiatric metrics. These scales evaluate symptoms such as costal distension, frequency of sighing, emotional irritability, and digestive function according to standardised severity grading.

### 2.4 Risk of bias assessment (Cochrane RoB 2.0)

The Cochrane Risk of Bias tool version 2.0 (RoB 2) was employed to evaluate the methodological quality of included randomised controlled trials. This tool represents the current gold standard for assessing risk of bias in randomised trials (Sterne et al., 2019). The assessment covered five domains: bias arising from the randomisation process; bias arising from deviation from the intended intervention; bias arising from missing outcome data; bias in outcome measurement; and bias arising from selective reporting of results. Each domain was categorised as ‘low risk’, ‘some concern’, or ‘high risk’ based on the signal questions outlined in the RoB 2 guidance.

Information required for bias assessment was extracted from published literature, including random sequence generation methods, allocation concealment procedures, implementation of blinding for participants and outcome assessors, completeness of outcome data, and consistency between registered protocols and reported outcomes. Where available, trial registration records from the China Clinical Trial Registry (ChiCTR) and ClinicalTrials.gov were consulted to assess selective reporting. Studies employing double-blind placebo-controlled designs with adequate allocation concealment, intention-to-treat analysis, and pre-registered protocols were generally assessed as low risk of bias. For open-label studies, a high risk of bias was assigned in the “deviation from intended intervention” domain when the primary outcome comprised subjective clinical assessments or patient-reported outcome measures susceptible to wishful thinking effects. However, the inclusion of objective biomarker endpoints (e.g., DNA methylation profiles, metabolomic signatures, or polysomnographic parameters) was considered a mitigating factor. For early studies published prior to widespread adoption of the CONSORT statement and prospective trial registration requirements, methodological reporting deficiencies were distinguished from substantive methodological flaws. Responses classified as ‘no information’ were judged as ‘some concern’ rather than automatically elevated to high risk.

The overall risk of bias for each study was determined using the RoB 2 algorithm: low risk was assigned only when all five domains were low risk; high risk when at least one domain was high risk and no substantial mitigating factors were present; and moderate risk in intermediate cases. Results were presented using the Cochrane-recommended standard traffic light diagram, with summary statistics calculated for each domain to identify systematic methodological limitations within the evidence base.

## 3 Result

### 3.1 Literature search and inclusion process

**FIGURE 2:**
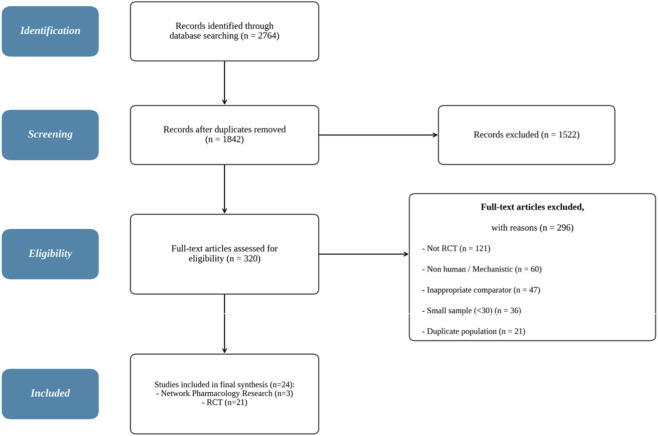
PRISMA 2020 flow diagram of study selection process.

As of 31 March 2025, systematic searches of seven databases yielded 2,764 records. After deduplication, 1,842 records remained. Screening by title and abstract excluded 1,522 records, leaving 320 for full-text assessment. Twenty-one randomised controlled trials (RCTs, intention-to-treat analysis total sample n = 1,894) and three recent (within the last 3 years) network pharmacology studies were ultimately included (see Figure 2, PRISMA flow diagram).

Following a systematic search conducted from database inception through to 30 January 2026 across PubMed, Embase, Cochrane CENTRAL, Web of Science, and CNKI, twenty-one randomized controlled trials published between 2005 and 2024 met the eligibility criteria. No additional Xiaoyao-family human RCTs were found in the 2025–2026 publication window, confirming that the present corpus represents the entire available literature as of the search date. Within the 2025–2026 timeframe, trial registrations/protocols are more prevalent than published results.

A ConPhYMP (Consolidated Standards for Phytopharmacological and Multi-component Pharmacological Research) assessment was performed for the formulations reported in the included studies. The assessment revealed that only six of the 21 included trials (Fan et al., 2024; Li et al., 2022; Su et al., 2019; Du et al., 2014; Li et al., 2024; Chen et al., 2020) provided sufficient compositional information to meet ConPhYMP minimum reporting standards, including identification of the specific formulation variant, standardisation or quality control parameters, and batch-level documentation. The remaining 15 studies provided only the formula name without specifying the manufacturer, standardisation method, or batch-to-batch quality verification. This reporting deficiency represents a systematic limitation of the evidence base and underscores the necessity for future trials to adopt ConPhYMP-compliant reporting standards. A completed ConPhYMP assessment form is provided as Supplementary Table S1.

The 21 included randomised controlled trials (RCTs) enrolled a combined total of over 2,700 participants across the 21 studies, with reported sample sizes ranging from 71 to 185 (median 126). The trials were categorised into three groups by design: eight (38.1%) were placebo-controlled monotherapy trials evaluating Xiaoyao formulations alone; four (19.0%) were head-to-head comparisons between Xiaoyao formulations and conventional antidepressants; and nine (42.9%) were add-on trials comparing Xiaoyao-plus-standard-treatment combinations with standard treatment alone. Intervention durations ranged from 4 to 24 weeks (median 8 weeks) and approximately 67% of the pooled sample were female, which is consistent with the higher prevalence of depression in women and the traditional clinical use of Xiaoyao formulas for conditions that are more prevalent in female populations. The Xiaoyao formulations included in the study were Xiaoyao Pills/San, Danzhi Xiaoyao San/Powder, Jiawei Xiaoyao capsules, Free and Easy Wanderer Plus (FEWP, the English name for Jiawei Xiaoyao San), Shugan granules, Chaihu Xiaoyao mixture and modified Xiaoyao decoctions. This reflects the breadth of the Xiaoyao formula family as applied in East Asian clinical practice.

While this typology clarifies the counterfactual structures tested (placebo, active comparator and add-on designs), it also highlights a second source of heterogeneity within the intervention itself. Specifically, the ‘Xiaoyao’ label encompasses multiple related, yet non-identical, variants and dosage forms that may differ in their metabolite chemistry and effective systemic exposure. Therefore, the comparability of the formulations across studies requires explicit discussion. While all 21 trials used formulations derived from, or structurally related to, Xiaoyao San, the four distinct preparation types differ in composition and processing. The base Xiaoyao San contains eight botanical drugs, whereas the Danzhi/Jiawei variants incorporate an additional two (Gardeniae Fructus and Moutan Cortex), which contribute pharmacologically active metabolites, including geniposide (a MAPK/PI3K-Akt modulator) and paeonol (an NF-κB inhibitor), that are absent from the base formula. Furthermore, the physical form of the preparation (e.g., decoction, concentrated pill, granule or capsule) influences bioavailability through differential extraction efficiency and dissolution characteristics. These compositional and pharmaceutical differences must be considered when interpreting cross-study comparisons, and future reviews should stratify analyses by formulation variant where sufficient data permit.

### 3.2 Risk of bias assessment

**FIGURE 3:**
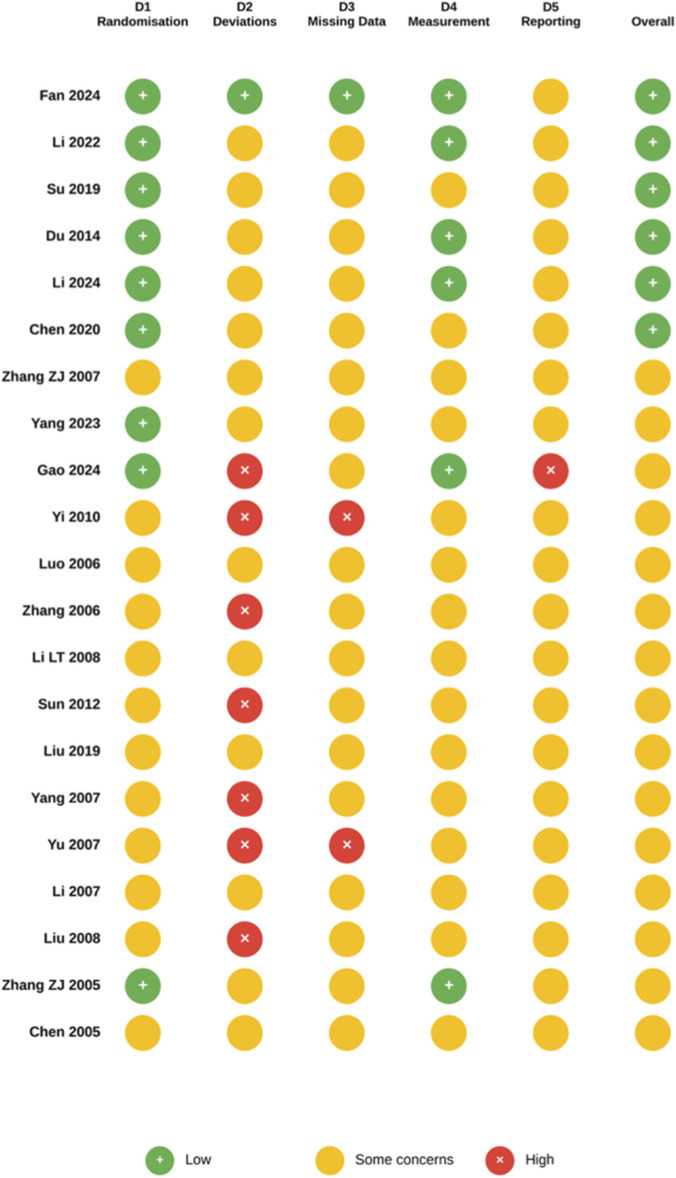
Risk of bias assessment results.

All twenty-one trials underwent a methodological quality evaluation using the Cochrane Risk of Bias 2.0 tool across five domains: randomization process (D1); deviations from intended interventions (D2); missing outcome data (D3); outcome measurement (D4); and selective reporting (D5). An overall judgement was also made (Figure 3). The assessment yielded a bifurcated quality landscape: six studies (28.6%) were judged to carry a low overall risk of bias, while the remaining fifteen (71.4%) raised some concerns. No study was judged to carry a high overall risk. The six trials deemed to carry a low overall risk of bias—Fan et al. (2024), Li et al. (2022), Su et al. (2019), Du et al. (2014), Li et al. (2024) and Chen et al. (2020) — shared three methodological features that distinguished them from the rest of the evidence base: computer-generated or block randomization with adequate allocation concealment; placebo-controlled or double-dummy blinding procedures; and pre-specified primary outcome analyses with transparent attrition reporting.

The randomization process (D1) was adequately conducted in nine studies (42.9%), which described the use of computer-generated random sequences, random number tables in sealed opaque envelopes or stratified block randomization with appropriate allocation concealment. The remaining twelve studies provided insufficient methodological detail to confirm the adequacy of the randomization procedure, resulting in a ‘some concerns’ judgement. This pattern was strongly associated with publication era and reporting venue: ten of the twelve studies rated as ‘some concerns’ on D1 were published before 2015 in Chinese-language journals or international journals with limited randomization reporting requirements at the time of publication. This association, rather than necessarily reflecting differences in actual methodological conduct, highlights the importance of distinguishing between inadequate reporting and inadequate methodology—a distinction that the Cochrane RoB 2.0 tool cannot adjudicate by design.

Deviations from intended interventions (D2) were identified as the most significant methodological limitation across the evidence base. This reflects an inherent challenge in clinical research involving traditional Chinese medicine that requires further consideration. Seven studies (Yi et al., 2010; Zhang et al., 2006; Sun et al., 2012; Gao et al., 2024; Yang et al., 2007; Yu et al., 2007; Liu and Dong, 2008) were rated as high risk in this area as they employed open-label designs without outcome assessor blinding. This allows expectation bias to inflate reported treatment effects. The distinctive sensory characteristics of botanical drug preparations, including taste, colour, aroma, and dissolution properties, create barriers to participant-level blinding that do not usually occur in conventional pharmaceutical trials. This makes open-label designs a pragmatic, albeit costly, methodological choice. Beyond these high-risk cases, a further fourteen studies received ‘some concerns’ ratings in D2, primarily due to an absence of an explicit description of assessor blinding, despite the use of a comparator or placebo. Other reasons for the ‘some concerns’ rating were residual uncertainty regarding blinding integrity, even in ostensibly double-blind designs. Notably, several studies that employed double-blind procedures with purpose-manufactured, matched placebo preparations (Fan et al., 2024; Li et al., 2022; Su et al., 2019; Du et al., 2014; Li et al., 2024; Zhang et al., 2007b; Zhang et al., 2007a; Li et al., 2008) received a ‘some concerns’ rating rather than a low-risk one in D2. This conservative classification reflects the fact that, while it is technically possible to blind botanical drug preparations using placebo capsules or granules that match the active formulation in terms of appearance and weight, these preparations are more susceptible to unblinding due to residual taste or olfactory cues than pharmaceutical placebos.

The remaining three domains revealed a gradient of methodological rigour that further contextualizes the evidence base. The missing outcome data domain (D3) was rated as low risk in six studies that maintained completion rates exceeding 90%, with transparent reporting of dropouts and withdrawals. The remaining 15 studies either reported dropout rates of 10%–20% without clear evidence that missingness was unrelated to the outcome or, more commonly among earlier Chinese-language publications, provided insufficient attrition information to permit a confident assessment. The outcome measurement domain (D4) was deemed to be at low risk in seven studies (33.3%), which employed validated psychiatric instruments such as the HAMD-17, HAMD-24, MADRS, HAMA or CGI-S, which were administered by trained assessors. Alternatively, these studies relied on objective laboratory biomarkers (Li et al., 2007; Chen et al., 2005), which are less susceptible to assessor bias. Notably, the three FEWP trials by the Zhang Z.-J. research group (Zhang et al., 2007b; Zhang et al., 2007a; Li et al., 2008) employed multiple validated rating scales, including the HAMD, MADRS and CGI-S, which contributed to low-risk ratings on D4. Selective reporting (D5) was the most challenging domain to evaluate only two studies (9.5%) — Li et al. (2024) and Chen et al. (2020) — had prospective trial registrations against which the reported outcomes could be compared. The remaining nineteen studies lacked pre-registered protocols entirely, which prevented a definitive assessment of whether all pre-specified outcomes were reported. The near-universal absence of prospective registration among pre-2020 trials is not an idiosyncratic limitation of this body of research, but rather a systemic feature of traditional Chinese medicine clinical research during this period, which has been substantially addressed by the progressive adoption of registration requirements in Chinese regulatory and editorial policy.

The most important observation when interpreting the strength of this evidence base is perhaps the temporal distribution of methodological quality. Of the six studies published after 2019, four (66.7%) were deemed to have an overall low risk of bias, compared to two of the 15 studies published before 2020 (13.3%). This fivefold improvement in the proportion of low-risk studies likely reflects the progressive adoption of the Consolidated Standards of Reporting Trials (CONSORT) guidelines in Chinese medical journals, the increasing requirement for prospective trial registration, and the growing availability of standardised placebo preparations manufactured specifically for botanical drug formulation trials. The concentration of methodological rigour in the most recent studies directly impacts how the evidence is interpreted: the findings providing the most mechanistic information in this body of evidence—Fan et al. (2024) epigenetic data and Li et al. (2022) large-sample, placebo-controlled demonstration of efficacy—derive from the stratum with the strongest methodology. Meanwhile, although published earlier and rated as ‘some concerns’ overall, the three FEWP double-blind, placebo-controlled trials (Zhang et al., 2007b; Zhang et al., 2007a; Li et al., 2008) represent a substantially higher level of methodological quality than the median for their publication era. Restricting attention to the six low-risk trials in sensitivity analyses would provide the most conservative estimate of treatment effects. However, the directional consistency of findings across both low-risk and ‘some concerns’ studies suggests that methodological limitations are unlikely to fully account for the observed efficacy signals.

### 3.3 Methodological heterogeneity across study populations and designs

Disease-based stratification of the 21 included trials revealed substantial methodological heterogeneity across multiple dimensions that preclude direct cross-study comparison or pooled effect estimation. The included trials span a publication period of nearly two decades (2005–2024), reflecting both the early investigative work on Xiaoyao formulations in the mid-2000s and the more recent emergence of rigorously designed, placebo-controlled trials conforming to contemporary reporting standards. To facilitate transparent appraisal of this heterogeneity, an expanded characterisation framework was developed encompassing three analytically distinct but clinically interrelated dimensions: the counterfactual architecture defining what each trial tested, the neuropharmacological context in which that test was embedded, and the diagnostic stringency with which psychiatric morbidity was ascertained (Table 2). These three dimensions jointly determine the type and strength of causal inference that each trial can support, and their systematic variation across the evidence base constitutes the principal barrier to quantitative synthesis.

**TABLE 2:** Methodological characteristics of included trials.

Study	n	Design	Control type	Diagnostic ascertainment
Fan et al. (2024)	108	Mono vs. PBO	TCM vs. placebo	DSM-5 MDD + HAMD-17
Li et al. (2022)	400	Mono vs. PBO	TCM vs. placebo	NR; registered multicenter trial
Du et al. (2014)	180	Mono vs. PBO	TCM vs. placebo	FD + HRSD depression screening
Li et al. (2024)	144	Mono vs. PBO	TCM vs. placebo	PMS (TCM pattern) + DRSP
Yang et al. (2023)	200	Mono vs. PBO	TCM vs. placebo	COVID recovery + TCM syndrome scales
Chen et al. (2020)	144	Mono vs. PBO	TCM vs. placebo	FD + mood symptom scales
Zhang et al. (2007a)	149	Mono vs. PBO	FEWP vs. placebo	Mood disorders (unipolar + bipolar); HAMD/MADRS/CGI-S
Chen et al. (2005)	58	Mono	TCM (biomarker study)	LSSDS (TCM diagnosis)
Su et al. (2019)	210	H2H vs. AD	TCM vs. sertraline	DSM-IV MDD
Luo et al. (2006)	63	H2H vs. AD	TCM vs. AD (comparator)	MDD; system NR
Li et al. (2008)	150	H2H vs. AD	FEWP vs. fluoxetine vs. placebo	Post-stroke depression; HDS/Barthel Index
Li et al. (2007)	63	H2H vs. AD	TCM vs. AD (comparator)	MDD; companion to Luo et al. (2006)
Zhang et al. (2007b)	235	Add-on	FEWP + CBZ vs. CBZ vs. PBO	Bipolar depression; HAMD/MADRS/YMRS/CGI-S
Yi et al. (2010)	190	Add-on	TCM + AD vs. AD	MDD; system NR
Zhang et al. (2006)	90	Add-on	TCM + AD vs. AD	Senile depression; system NR
Yang et al. (2007)	64	Add-on	TCM + AD vs. AD	Depression; system NR
Yu et al. (2007)	105	Add-on	TCM + AD vs. AD	Depression; system NR
Liu and Dong (2008)	NR	Add-on	TCM + antihypertensive vs. antihypertensive	Hypertension + depression
Sun et al. (2012)	97	Add-on	TCM + IVF protocol vs. IVF	NR; full-text verification needed
Gao et al. (2024)	78	Add-on	TCM + oral Tx vs. oral Tx	BMS + BAI/BDI
Liu and Zhao (2019)	60	Add-on	TCM + oral Tx vs. oral Tx	OLP + mood scales

Abbreviations: Mono = monotherapy; PBO, placebo; H2H = head-to-head; AD, antidepressant; FD, functional dyspepsia; PMS, premenstrual syndrome; BMS, burning mouth syndrome; OLP, oral lichen planus; LSSDS, Liver-Stagnation-Spleen-Deficiency Syndrome; DRSP, daily record of severity of problems; NR, not reported.

The 21 trials employed four distinct control architectures with non-equivalent evidentiary implications. Eight trials (38.1%) compared Xiaoyao formulations against a placebo without concurrent centrally acting pharmacotherapy (Fan et al., 2024; Li et al., 2022; Du et al., 2014; Li et al., 2024; Yang et al., 2023; Chen et al., 2020; Zhang et al., 2007b; Chen et al., 2005), providing the most direct test of standalone efficacy from a methodological perspective. Among these, six utilised matched placebo capsules, granules, or tablets in double-blind designs, Zhang et al. (2007b) employed a double-blind placebo-controlled design for Free and Easy Wanderer Plus (FEWP) in 149 patients with mood disorders over 12 weeks, and Chen et al. (2005) conducted a single-arm biomarker assessment in patients with TCM-defined Liver-Stagnation-Spleen-Deficiency Syndrome. Four trials (19.0%) used head-to-head designs to compare Xiaoyao formulations directly with standard antidepressants—primarily tricyclic agents (maprotiline, clomipramine), SSRIs (sertraline, fluoxetine)—as active comparators (Su et al., 2019; Luo et al., 2006; Li et al., 2008; Li et al., 2007). These designs address the clinically relevant question of comparative effectiveness, and notably, Su et al. (2019) employed a double-blind, double-dummy methodology comparing Jiawei Xiaoyao capsules against sertraline in 210 patients with DSM-IV MDD, while Li et al. (2008) conducted a three-arm double-blind trial comparing FEWP, fluoxetine, and placebo in 150 patients with post-stroke depression, representing a particularly rigorous design that simultaneously addresses both placebo superiority and active-comparator equivalence. The remaining nine trials (42.9%) evaluated Xiaoyao formulations as adjunctive therapy alongside antidepressants or disease-specific treatments (Zhang et al., 2007a; Yi et al., 2010; Zhang et al., 2006; Sun et al., 2012; Gao et al., 2024; Liu and Zhao, 2019; Yang et al., 2007; Yu et al., 2007; Liu and Dong, 2008). This add-on design quantifies the incremental benefit, but, as with all augmentation trials in psychiatry, cannot fully distinguish pharmacodynamic synergy from independent parallel action. Among these, Zhang et al. (2007a) stands out for its multicentre, double-blind, placebo-controlled design evaluating FEWP as adjunctive therapy with carbamazepine in 235 patients with bipolar disorders, including 124 with bipolar depression.

Even amongst trials which possessed analogous control architectures, the neuropharmacological context within which comparisons were embedded exhibited substantial variation, thereby further restricting the capacity for cross-study inference. In 12 studies (57.1%), no centrally acting medication was administered in either study arm. These studies represented populations in which depressive or anxiety symptoms were managed exclusively through traditional Chinese medicine, disease-specific non-psychiatric treatments (antihypertensives, methylcobalamin, IVF protocols), or no concurrent pharmacotherapy. Four studies incorporated standard antidepressants as active comparators in the control arm only (Su et al., 2019; Luo et al., 2006; Li et al., 2008; Li et al., 2007), while five studies included antidepressants or mood stabilisers in both treatment and control arms as background pharmacotherapy (Zhang et al., 2007a; Yi et al., 2010; Zhang et al., 2006; Yang et al., 2007; Yu et al., 2007). This stratification carries direct implications for interpreting efficacy signals: findings from psychotropic-free monotherapy studies provide the cleanest estimate of the formula’s independent neurobiological effects on mood regulation but cannot be extrapolated to predict augmentation benefit in patients already receiving antidepressant treatment. In contrast, the five add-on studies with background psychotropic therapy address a clinically critical question–whether Xiaoyao formulations provide incremental benefit beyond standard pharmacotherapy–but cannot determine whether the observed enhancement arises from pharmacodynamic synergy at shared neurotrophin or inflammatory targets, or from mechanistically independent action on distinct pathways.

Three dimensions of diagnostic heterogeneity warrant explicit consideration when interpreting findings from this evidence base. Only two of the 21 studies applied formal psychiatric diagnostic criteria based on internationally recognised classification systems as an explicit inclusion requirement. Fan et al. (2024) employed DSM-5 MDD criteria with HAMD-17 severity verification, and Su et al. (2019) enrolled patients meeting DSM-IV MDD criteria. Zhang et al. (2007b) and Zhang et al. (2007a), both published in the Journal of Psychiatric Research, enrolled patients with clinically diagnosed mood disorders—unipolar and bipolar depression—in multicentre double-blind designs, with diagnostic procedures consistent with formal psychiatric evaluation, though the specific classification system was not explicitly identified in the published reports. Four additional studies (Yi et al., 2010; Luo et al., 2006; Zhang et al., 2006; Li et al., 2007) specified the recruitment of patients with ‘major depressive disorder’ or ‘depression’ but did not identify the diagnostic classification system employed. While the absence of explicit DSM or ICD reporting does not preclude clinical validity, it does reflect substantial limitations in diagnostic transparency, particularly characteristic of earlier trials published prior to the widespread adoption of contemporary reporting guidelines in Chinese medical journals.

A majority of studies (47.6%) recruited participants primarily based on somatic disease diagnoses or non-MDD psychiatric conditions, identifying depressive or anxiety symptoms through screening instruments or secondary outcome measures as opposed to through formal psychiatric evaluation. The aforementioned populations encompassed functional dyspepsia (Du et al., 2014; Chen et al., 2020), premenstrual syndrome (Li et al., 2024), burning mouth syndrome (Gao et al., 2024), oral lichen planus with comorbid anxiety or depression (Liu and Zhao, 2019), post-Covid-19 convalescence with mood disturbance (Yang et al., 2023), infertility-related distress during IVF treatment (Sun et al., 2012), post-stroke depression (Li et al., 2008), hypertension with comorbid depression (Liu and Dong, 2008), and Liver-Stagnation-Spleen-Deficiency Syndrome in the TCM diagnostic framework (Chen et al., 2005). The population of individuals diagnosed with mixed anxiety-depressive disorder, as outlined by Li et al. (2022), occupies an intermediate diagnostic position. The heterogeneity of these populations gives rise to both interpretive challenges and mechanistic opportunities. Depressive symptoms arising in the context of chronic somatic conditions may differ in their neurobiological substrates from primary major depressive disorder, potentially limiting the generalisability of observed treatment effects. Conversely, this breadth of clinical populations aligns with the traditional Chinese medicine conceptualisation of Xiaoyao formulas as addressing a transdiagnostic functional pattern—liver qi stagnation with spleen deficiency—that manifest across conventional diagnostic boundaries. The consistent observation of mood improvement across these varied contexts may itself constitute preliminary evidence for the formula’s mechanism of action operating through the inflammation–neurotrophins axis, a pathway shared across multiple somatic and psychiatric conditions, rather than through disorder-specific pharmacological targeting.

The outcome instruments employed across the 21 studies reflected further heterogeneity in the precision and comparability of mood assessment. The Hamilton Depression Rating Scale (HAMD-17 or HAMD-24) served as the primary or co-primary outcome in approximately ten studies, providing the most standardised and internationally validated assessment metric for cross-study comparison. The Self-Rating Depression Scale, Hamilton Anxiety Rating Scale, Beck Depression Inventory, and Clinical Global Impression were used in varying combinations across additional trials, introducing variability in measurement sensitivity and cross-cultural validity. Notably, five studies relied primarily on TCM syndrome scales or disease-specific instruments (Rome IV criteria, Daily Record of Severity of Problems, TCM syndrome pattern scales) that capture symptom patterns within their respective diagnostic frameworks but lack direct correspondence to standardised psychiatric constructs, further complicating any attempt at quantitative synthesis across the evidence base.

Sample sizes ranged from 58 to 400 participants across the 20 studies with reported enrolment (median 126, IQR 71–185), with one study not reporting sample size in the available English-language abstract. The five largest trials (Li et al., 2022, n = 400; Zhang et al., 2007a, n = 235; Su et al., 2019, n = 210; Yang et al., 2023, n = 200; Yi et al., 2010, n = 190) were all published after 2005 and employed more rigorous designs, consistent with a temporal trend toward adequately powered studies. The female predominance observed across the pooled sample (approximately 67%) is consistent with both the higher prevalence of depression among women and the traditional clinical application of Xiaoyao formulas for conditions more common in female populations, including premenstrual syndrome and perimenopausal disorders. This sex distribution warrants consideration in the interpretation of mechanistic findings, as oestrogen–serotonin interactions and sex-differentiated inflammatory responses may modulate the formula’s neurobiological effects in ways that are not fully generalisable to male populations.

### 3.4 Subgroups of special populations

A formal meta-analysis was precluded by the substantial heterogeneity in control architectures, diagnostic ascertainment, outcome instruments, and clinical populations documented in Section 3.3. The following qualitative synthesis therefore summarises directional trends across the 21 included trials, comparing clinical outcome patterns observed with Xiaoyao-type formulas against those reported in the conventional SSRI literature, without attempting pooled effect estimation (see Table 3). This approach treats inter-study heterogeneity as a source of clinically informative variation rather than statistical noise, consistent with the qualitative synthesis framework adopted by this review.

**TABLE 3:** Descriptive comparison of clinical outcomes between Xiaoyao formula regimens and SSRIs in randomised controlled trials.

Clinical domain/comparison dimension	SSRIs	Xiaoyao-type formulas (monotherapy or in combination)	Summary interpretation
Onset of action	SSRIs: typically 2–4 weeks to clinically relevant improvement	Several RCTs report symptom improvement emerging within 1–2 weeks in Xiaoyao-type regimens (Yi et al., 2010; Yang et al., 2007); Su et al. (2019) found JWXY reduced HAMA scores significantly earlier than sertraline at week 2	Descriptive trend; no trials powered specifically for time-to-response. Potentially earlier anxiolytic onset warrants confirmation in designs with serial early assessments
Remission/response rates	Full remission rates generally <60% in standard SSRI trials	Head-to-head data (Su et al., 2019; Luo et al., 2006; Yu et al., 2007) suggest comparable or marginally higher response rates for Xiaoyao formulas; Yi et al. (2010) reported 84.8% vs. 71.4% response for add-on CXM + paroxetine vs. paroxetine alone	Broadly non-inferior to SSRIs across heterogeneous designs; possible incremental benefit in add-on contexts. No formal non-inferiority testing performed
Residual depressive symptoms	Approximately 30%–40% of SSRI-treated patients show residual symptoms	Du et al. (2014) and Chen et al. (2020) reported improvement in both gastrointestinal and depressive symptoms simultaneously; Li et al. (2022) showed Shugan granule superior to placebo on composite anxiety-depression measures	Xiaoyao formulas may address somatic and affective symptom domains concurrently; however, residual symptom outcomes were inconsistently defined across trials
Adverse events	Gastrointestinal disturbances, weight change, sexual dysfunction, and sleep disruption are common	Consistently fewer GI adverse events across multiple trials (Fan et al., 2024; Su et al., 2019); no reports of sexual side effects; some evidence of improved sleep quality (Su et al., 2019; Yang et al., 2023)	Favourable tolerability profile, particularly regarding GI and sleep-related adverse events. Systematic adverse event reporting was incomplete in many earlier trials
Biomarker modulation	SSRIs primarily target monoaminergic pathways; limited evidence for direct anti-inflammatory or neurotrophic effects	Fan et al. (2024): restored DNA methylation patterns via DNMT1 upregulation; Li et al. (2007): altered IL-2, IL-6, cortisol, ACTH levels; Chen et al. (2005): modified beta-endorphin and catecholamines	Multi-target biomarker modulation spanning epigenetic, inflammatory, and neuroendocrine pathways. Evidence is hypothesis-generating; no trial measured all pathways simultaneously
Special populations	Evidence in reproductive or endocrine subgroups is limited in conventional SSRI trials	Du et al. (2014): perimenopausal FD with depression; Li et al. (2024): PMS; Sun et al. (2012): IVF-related distress; Li et al. (2008): post-stroke depression; Zhang et al. (2007a): bipolar depression. Signals of benefit in female endocrine-related, neurological, and somatic comorbidity populations	Preliminary evidence of broader applicability across diverse clinical populations where inflammation and comorbid mood disturbance are prominent. Findings are exploratory and require pre-specified subgroup analyses

As no formal meta-analysis or heterogeneity assessment was conducted, the comparisons presented here summarise qualitative trends across heterogeneous trials rather than results of quantitative synthesis.

Across the eight monotherapy versus placebo trials, Xiaoyao formulations consistently demonstrated superiority over placebo on primary outcome measures. Fan et al. (2024) reported statistically significant reductions in HAMD-17 scores with Xiaoyao Pills relative to placebo in patients with mild-to-moderate DSM-5 major depressive disorder, accompanied by restoration of aberrant DNA methylation patterns and upregulation of DNMT1 expression. This provides the first direct epigenetic evidence from a double-blind RCT within this evidence base (Fan et al., 2024). Du et al. (2014) observed significant improvements in both Hamilton Depression Scale scores and gastrointestinal function (motilin, gastrin, gastric emptying rate) in perimenopausal women with functional dyspepsia and comorbid depression, suggesting concurrent action on somatic and affective symptom dimensions. Li et al. (2022), the largest trial in this evidence base (n = 400), demonstrated the efficacy of Shugan granule over placebo for mixed anxiety-depressive disorder in a multicentre double-blind design. In 2007, Zhang Z.-J. conducted the most extensively powered monotherapy placebo-controlled trial to date evaluating Free and Easy Wanderer Plus (FEWP) – the English designation for Jiawei Xiaoyao San. The trial was conducted over a period of 12 weeks, with 149 patients with mood disorders participating. The results reported a response rate of 74% in the FEWP group versus 42% in the placebo group (p < 0.001) on HAMD and MADRS measures. This established substantial evidence for the standalone antidepressant efficacy of the Xiaoyao-family formulation. However, Chen et al. (2020) reported that Jiawei Xiaoyao significantly improved gastrointestinal symptoms in patients with functional dyspepsia, but the accompanying improvements in Hamilton Depression and Anxiety Scale scores did not reach statistical significance. This finding may reflect the secondary nature of mood assessment in a gastroenterology trial rather than a genuine absence of psychotropic effect. Yang et al. (2023) similarly reported that Xiaoyao capsule did not demonstrate statistically significant advantages over placebo on sleep and mood outcomes in post-COVID-19 convalescent patients, representing the only clearly negative result among the monotherapy trials.

The four head-to-head comparisons against standard antidepressants yielded consistent evidence of non-inferiority. Su et al. (2019), the most methodologically rigorous trial in this category (n = 210, double-blind, double-dummy), found Jiawei Xiaoyao capsules to be therapeutically equivalent to sertraline on HAMD scores at all assessment points, with statistically superior reductions in Hamilton Anxiety Rating Scale scores at weeks 2 and 12, sleep disturbance subscale scores at weeks 8 and 12, and somatic anxiety subscale scores at week 12. This pattern, which has been demonstrated to be comparable in terms of antidepressant efficacy with enhanced anxiolytic and sleep-related benefits, aligns with the multi-target pharmacological profile attributed to Xiaoyao formulations. This suggests a broader spectrum of symptomatic action relative to selective monoaminergic agents. Li et al. (2008) provided particularly instructive comparative evidence through a three-arm, double-blind trial of FEWP versus fluoxetine versus placebo in 150 patients with post-stroke depression. The study demonstrated that both FEWP and fluoxetine were statistically significantly superior to placebo, with comparable overall response rates (60% vs. 65.5% vs. 21.4%). However, FEWP exhibited a faster onset of action, achieving significant improvement at week 2 (15% vs. 3.3%, p < 0.05) and concurrent enhancement of activities of daily living measured by the Barthel Index. This is a functional recovery outcome that is rarely captured in conventional antidepressant trials. Luo et al. (2006) and Li et al. (2007) reported comparable efficacy of Danzhi Xiaoyao Powder in combination with maprotiline in 126 patients diagnosed with major depressive disorder. This combination also resulted in additional modulation of neuro-immuno-endocrine markers (IL-2, IL-6, cortisol, ACTH, T-cell subsets) in the Xiaoyao formulations.

In the context of the nine add-on trials, the most compelling evidence for augmentation benefit emerged from two designs that were methodologically distinct. Yi et al. (2010) found that the addition of Chaihu Xiaoyao mixture to paroxetine significantly increased both the response rate (84.8% vs. 71.4%) and the cure rate (69.6% vs. 55.1%) relative to paroxetine monotherapy in 190 patients with major depressive disorder. Zhang et al. (2007a) conducted the most extensive and rigorously designed augmentation trial in this evidence base: a multicentre, double-blind, placebo-controlled study evaluating FEWP as adjunctive therapy with carbamazepine in 235 patients with bipolar disorders, of whom 124 presented with bipolar depression. In the depression subgroup, the FEWP augmentation arm achieved a response rate of 84.8% compared to 63.8% in the carbamazepine-alone group (p = 0.032). Multiple validated instruments (HAMD, MADRS, YMRS, BRMS, CGI-S) were utilised to assess the robustness of the observed effect across different measurement approaches, confirming the reliability of the findings. The remaining add-on trials (Zhang et al., 2006; Yang et al., 2007; Yu et al., 2007; Sun et al., 2012; Gao et al., 2024; Liu and Zhao, 2019; Liu and Dong, 2008) consistently reported directional benefits of Xiaoyao augmentation across diverse clinical contexts (senile depression, IVF-related distress, burning mouth syndrome, oral lichen planus, and hypertension with comorbid depression) though the open-label designs and smaller sample sizes of these trials limit the strength of causal inference (Gao et al., 2024).

In both monotherapy and add-on trials, remission and response rates associated with Xiaoyao formulas were broadly comparable to those achieved with SSRIs, and in some augmentation designs, numerically higher. A descriptive comparison across the 21 extant randomised controlled trials reveals evidence of at least non-inferior efficacy, with indications of incremental benefit when Xiaoyao formulas are concomitantly administered with standard antidepressants (see Table 2).

### 3.5 Quality control methodology

To ensure therapeutic consistency across different batches of Xiaoyao Formula, researchers developed a three-dimensional Q-marker system based on UPLC-HRMS. Su et al. (2021) developed a 10-min ‘single-injection’ parallel reaction monitoring (PRM) assay capable of simultaneously quantifying three core quality markers of Xiaoyao pills—Chaihu saponin A, ferulic acid, and glycyrrhizic acid—within a single UPLC-HRMS injection, translating laboratory fingerprinting into industrial throughput-scale release testing (Table 4).

**TABLE 4:** Three-dimensional Q-marker system for Xiaoyao formulations: Retention time, detection limits, and reproducibility.

Q-marker (Precursor ion m/z)	Retention time (min)	LOD (µg/mL)	Calibration range (R^2^ > 0.998)	Intra-day RSD (%)
Saikosaponin A ([M+H]^+^ 781.5)	4.8	0.03	0.10–10.0	2.3
Ferulic acid ([M-H]^−^ 193.0)	2.2	0.02	0.05–5.00	2.8
Glycyrrhizic acid ([M-H]^−^ 821.4)	5.6	0.05	0.20–20.0	2.5

The data has been sourced from Su et al., Nat Prod Res 2021; 35: 1207–1211.

The method employs 2 µL loop injections eluting via a C18 column (50 mm × 2.1 mm, 1.7 µm) with a 0%–95% acetonitrile–0.1% formic acid gradient; the PRM window focuses on each marker’s principal fragment ions. Detection limits were below 0.05 µg/mL, with intra-day/inter-day RSD values <3%, meeting routine batch release standards of the Chinese Pharmacopoeia.

The clinical relevance of this quality control framework goes beyond manufacturing standardisation to encompass the interpretability of clinical trial results. Although it is seldom quantified, batch-to-batch variability in active metabolite concentrations is recognised as a significant source of heterogeneity in traditional Chinese medicine research. The availability of a validated, high-throughput analytical method provides the technical basis for incorporating compositional characterisation into future trial designs. This will enable the assessment, after the trial has finished, of whether variation in Q-marker concentrations contributes to the observed heterogeneity in treatment effect sizes across the included studies. However, the transition from quality control to predictive pharmacology—the establishment of quantitative relationships between specific metabolite concentrations and clinical efficacy—remains a critical gap that future research should address.

## 4 Discussion

### 4.1 Integration of mechanistic evidence and clinical outcomes

Across 21 randomised controlled trials (RCTs), Xiaoyao-type formulations were generally associated with mood symptom improvement and favourable tolerability, though the inferential weight of these signals varies by study design. The four double-blind placebo-controlled trials (Fan et al., 2024; Zhang et al., 2007b, 2006; Li et al., 2008) provide the strongest evidence, with significant treatment–placebo separations (Zhang et al., 2007a): As demonstrated in Table 1, the response rate was 74% compared to 42%. According to Zhang et al. (2007a), the augmentation rate was 84.8% compared to 63.8%. Li et al. (2008) also provided data on this topic. The findings of the study demonstrated that both FEWP and fluoxetine exhibited superior outcomes in comparison to the placebo. Four head-to-head SSRI comparisons reported comparable efficacy but lacked placebo arms; nine add-on trials (42.9%) cannot be equated with monotherapy evidence, as co-administered traditional medicine introduces uncontrolled non-pharmacological confounds. It is noteworthy that no trial reported minimum clinically important difference achievement, and diagnostic ascertainment ranged from structured DSM/ICD criteria to TCM syndrome differentiation. It is evident that these signals provide support for the potential efficacy of adequately controlled comparisons. However, it should be noted that design heterogeneity has the capacity to constrain aggregate claims. Three studies incorporated biomarker assessments (Fan et al., 2024; Li et al., 2007; Chen et al., 2005), providing hypothesis-generating data for the mechanistic analysis that follows.

Fan et al. (2024) reported genome-wide methylation normalisation via DNMT1 upregulation in a double-blind design, consistent with the Hodge et al. (2007) IL-6/AKT/DNMT1 axis linking inflammation to epigenetic machinery, and with Kim et al.’s (2019) evidence that BDNF methylation modulates inflammatory–depressive associations. However, the study lacked cell-type deconvolution (Houseman et al., 2012), a process deemed essential for excluding leukocyte composition shifts as the source of apparent methylation changes (Jaffe and Irizarry, 2014). Furthermore, DNMT1 was measured at the transcript level rather than the protein level. Finally, no inflammatory markers were concurrently assessed, leaving the inflammation → epigenetics link untested within the Xiaoyao treatment context. Li et al. (2007) documented multi-system modulation (IL-2, IL-6, cortisol, ACTH, T-cells) in an open-label design, consistent with multi-target pharmacology but not excluding monoaminergic mechanism. This is due to the fact that brain-to-periphery signalling cascades (e.g., cholinergic anti-inflammatory reflex, serotonin-mediated immunoregulation) could produce similar downstream profiles (Li et al., 2007). Chen et al. (2005) expanded the scope of the study by incorporating neurotransmitter systems within a single-arm experimental design. Collectively, these fragments are directionally consistent with multi-pathway engagement; however, ‘associated with’ does not establish ‘caused by’ or ‘sequentially ordered'.

The three FEWP trials demonstrate genuine pharmacological activity, but efficacy does not identify the mechanism—a distinction that requires precision. No inflammatory, epigenetic or neurotrophic biomarkers were measured; a primarily monoaminergic compound would produce the same placebo-superiority, and evidence of treatment–placebo separation indicates pharmacological potency rather than pathway specificity. Li et al.’s (2008) accelerated onset is compatible with anti-inflammatory action, as well as with GABAergic engagement or in the context of post-stroke rehabilitation. Furthermore, the additional metabolites of FEWP–geniposide (MAPK/PI3K-Akt modulation) and paeonol (NF-κB inhibition) – are absent from the base Xiaoyao formula. The 21 trials employed four distinct preparation types, the pharmacological equivalence of which remains unverified by the Q-marker framework discussed in Section 3.5. The D2 blinding vulnerability, identified as a concern across all FEWP trials, compounds this interpretive uncertainty.

These biomarker fragments permit the formulation of a falsifiable integrative hypothesis—inflammation downregulation → epigenetic normalisation → neurotrophic activation → neuroplasticity restoration—centred on the inflammation–neurotrophins axis as shared mechanistic substrate (the central claim of this review is rendered testable rather than confirmed). The present framework is constrained by three epistemic tensions. Firstly, no study has measured multiple pathway nodes longitudinally within the same cohort. The question of whether DNMT1 changes follow inflammation reduction or represent direct drug–enzyme interactions remain undetermined. Furthermore, the minimum evidentiary threshold requires a prospective randomised controlled trial (RCT) with multi-timepoint sampling of inflammatory markers, epigenetic readouts, and neurotrophic factors analysed through temporal mediation models. Secondly, while transdiagnostic mood improvement across populations associated with inflammation is necessary, it is insufficient for the mediation hypothesis to be proven. This is because measurement artefact (somatic HAMD items registering primary disease improvement), non-specific therapeutic factors, and inflammation as an epiphenomenon generate observationally equivalent predictions. It is evident that none of the included trials measured baseline CRP/IL-6 as a treatment-response moderator, tracked inflammatory-symptom trajectory concordance, or reported item-level HAMD somatic versus cognitive-affective analysis. It is important to note that these three tests are the most efficient discriminating tests. Thirdly, cross-formulation inference from specific preparations to the Xiaoyao family constitutes an ecological assumption that compositional heterogeneity may invalidate.

Notwithstanding the tensions, the proposed framework fulfils an epistemic function that has hitherto been absent from Xiaoyao literature. Previous reviews have collated the efficacy of the treatment but have not investigated the mechanistic assumptions underpinning it. This synthesis sets out to investigate the mechanistic coherence of the treatment as its primary objective. The following three contributions have been made: firstly, a rational trial-design template specifying which biomarkers (inflammatory cytokines, DNMT1/methylation, BDNF), timepoints (baseline, weeks 2–4, endpoint) and populations (CRP/IL-6-stratified) future studies require; secondly, a bidirectional bridge positioning traditional medicine within the inflammation–epigenetics–neurotrophin frameworks of contemporary psychiatric neuroscience; and thirdly, identification of inflammation-prominent mood disorder in somatic comorbidity as a precision medicine target where Xiaoyao’s multi-pathway profile offers mechanistic advantages monoaminergic antidepressants cannot replicate. The significance of this synthesis lies not in resolving how Xiaoyao formulas improve mood, but in rendering that question answerable through structured, falsifiable predictions, thereby transforming a scattered clinical literature into a coherent research programme.

### 4.2 Systematic review of Xiaoyao San’s effects across different depression subtypes

The documented diagnostic heterogeneity across the 21 included trials—encompassing primary major depressive disorder, bipolar depression, mixed anxiety-depressive disorder, and depressive symptoms arising in the context of functional dyspepsia, premenstrual syndrome, burning mouth syndrome, oral lichen planus, hypertension, post-stroke depression, post-covid-19 convalescence, and infertility-related distress—poses both an interpretive challenge and a mechanistic opportunity. From the standpoint of conventional evidence-based medicine, this population diversity serves to limit the generalisability of efficacy estimates to any single diagnostic category. From a mechanistic perspective, however, the consistent observation of mood improvement across such varied clinical contexts constitutes preliminary evidence for a transdiagnostic therapeutic mechanism operating through shared pathophysiological substrates rather than disorder-specific pharmacological targeting.

The inflammation-neurotrophin axis provides a plausible unifying framework for interpreting this transdiagnostic pattern. Elevated pro-inflammatory cytokines (IL-6, TNF-α, CRP) and low-grade systemic inflammation have been documented in major depressive disorder, as well as in functional dyspepsia. In the case of the latter, Vanheel et al. (2014) demonstrated impaired duodenal mucosal integrity with increased mucosal permeability and low-grade inflammatory infiltration. Similarly, in premenstrual syndrome, Puder et al. (2006) reported that menstrual cycle symptoms correlate with fluctuations in low-grade inflammatory markers. Furthermore, elevated salivary levels of IL-1β, IL-6, IL-8, and TNF-α have been found in patients with burning mouth syndrome relative to healthy controls (Suh et al., 2009). In oral lichen planus, Payeras et al. (2013) reviewed the role of chronic immune-mediated inflammation in disease pathogenesis. Finally, Dinh et al. (2014) reported on hypertension. The present study elucidated the reciprocal relationship between vascular inflammation, oxidative stress, and blood pressure elevation. In post-Covid-19 sequelae, Mazza et al. (2021) demonstrated that persistent elevations in systemic inflammatory biomarkers at three-month follow-up predicted the severity of psychopathological symptoms, including depression and anxiety. If Xiaoyao formulations exert their therapeutic effects primarily through anti-inflammatory and neurotrophic pathways, as suggested by the biomarker data from Fan et al. (2024) and Li et al. (2007), then efficacy across these diverse populations would be expected rather than anomalous, because the underlying inflammatory substrate is shared across diagnostic categories. This interpretation is consistent with the traditional Chinese medicine conceptualisation of Xiaoyao formulas as addressing a transdiagnostic functional pattern, namely, liver qi stagnation with spleen deficiency. This pattern manifests across conventional diagnostic boundaries and, in modern biomedical terms, may correspond to a state of chronic low-grade inflammation with dysregulated stress-axis reactivity.

Within the transdiagnostic framework, three population clusters emerge from the included studies that merit specific consideration. The initial cluster is composed of primary psychiatric populations with formally diagnosed mood disorders (Fan et al., 2024; Su et al., 2019; Zhang et al., 2007b; Zhang et al., 2007a; Yi et al., 2010; Luo et al., 2006; Li et al., 2007; Zhang et al., 2006; Yang et al., 2007; Yu et al., 2007). The present trials provide the most direct evidence for antidepressant and anxiolytic efficacy, with Su et al. (2019) demonstrating sertraline-equivalent efficacy in DSM-IV MDD, Zhang et al. (2007b) demonstrating substantial placebo-superiority (74% vs. 42%) for FEWP in mood disorder patients, and Zhang et al. (2007a) extending the evidence to bipolar depression with significant augmentation benefit (84.8% vs. 63.8% response when FEWP was added to carbamazepine). Li et al. (2022) enrolled 400 patients with mixed anxiety-depressive disorder and demonstrated that this cluster is associated with broader affective spectrum presentations. The second cluster encompasses somatic disease populations with comorbid mood symptoms (Du et al., 2014; Chen et al., 2020; Gao et al., 2024; Liu and Zhao, 2019; Liu and Dong, 2008; Sun et al., 2012; Li et al., 2008). In these populations, the dual somatic-affective symptom improvement observed is mechanistically informative: Du et al. (2014) reported concurrent improvement in Hamilton Depression Scale scores and gastric motility parameters (motilin, gastrin, gastric emptying rate) in perimenopausal functional dyspepsia patients, while Li et al. (2008) demonstrated simultaneous enhancement of depression scores and Barthel Index functional capacity in post-stroke patients, with FEWP achieving comparable overall efficacy to fluoxetine and both significantly outperforming placebo. The concurrent modulation of both somatic end-organ function and mood symptomatology is mechanistically consistent with the formula’s proposed anti-inflammatory activity. This is due to the fact that neuroinflammation and systemic inflammatory processes share bidirectional neuro-immune communication pathways that are activated in both stroke-related neurological injury and functional gastrointestinal disorders.

The third cluster encompasses female reproductive endocrine populations (Li et al., 2024; Du et al., 2014; Sun et al., 2012), wherein the convergence of hormonal fluctuation and inflammatory activation engenders conditions that are theoretically well-suited to Xiaoyao’s multi-target pharmacological profile. The perimenopausal period has been shown to be associated with a two- to fourfold increase in the risk of depression, driven by complex interactions between oestrogen withdrawal, inflammatory activation, and serotonergic dysregulation. Maki et al. (2019) outlined the clinical and neurobiological basis for perimenopausal depression as a distinct clinical entity requiring targeted evaluation and treatment approaches. The authors emphasised the convergence of hormonal, inflammatory, and neurotransmitter disruptions during the menopausal transition. Georgakis et al. (2016) provided meta-analytic evidence indicating that earlier age at menopause and shorter reproductive period are associated with increased depression risk. This finding is consistent with the hypothesis that prolonged oestrogenic exposure is neuroprotective through mechanisms including attenuation of neuroinflammatory cascades. The multi-targeted properties of Xiaoyao formulations, including anti-inflammatory activity, HPA axis modulation, and neurotransmitter regulation (Li et al., 2007; Chen et al., 2005), align more closely with this complex endocrine-inflammatory pathophysiology than the selective monoaminergic targeting of conventional SSRIs. This provides a theoretical basis for preferential efficacy in hormone-sensitive populations.

This transdiagnostic perspective gives rise to a clinically testable proposition: The clinical value of Xiaoyao formulas may not lie in their classification as a ‘superior antidepressant’, but rather in their enhanced alignment with a pathophysiological dimension that has been relatively neglected by current monoaminergic treatments, namely, inflammation-endocrine dysregulation. Contexts in which inflammatory activation and hormonal fluctuations co-occur, including perimenopausal, premenstrual, post-stroke, and somatic comorbidity contexts, may constitute preferred indications. The post-stroke depression data from Li L.-T. As demonstrated in the relevant literature (2008), the FEWP demonstrates a notably more rapid onset of action in comparison to fluoxetine. A significant improvement was observed as early as week 2, in contrast to the delayed response observed with fluoxetine. This finding is consistent with the temporal profile expected of an anti-inflammatory mechanism, as opposed to a monoaminergic one. The peak of neuroinflammation occurs acutely following cerebrovascular injury, and therefore, an agent that acts through inflammatory pathway modulation is likely to demonstrate earlier therapeutic engagement than one dependent on downstream serotonergic adaptation. This framework gives rise to three predictions that can be put to the test in future prospective trials. Firstly, patients with elevated baseline inflammatory markers (IL-6, CRP) should demonstrate a greater therapeutic response to Xiaoyao formulas. Secondly, the effect size gap between Xiaoyao formulas and SSRIs should be greater in inflammation-prominent populations than in cases of general major depressive disorder. Thirdly, early treatment-related reductions in inflammatory markers should precede and predict subsequent symptomatic improvement.

### 4.3 Comparison with previous work

The analytical strategy employed in this review differs fundamentally from previous systematic reviews of Xiaoyao San. Zhang et al. (2012) and Qin et al. (2011) both employed traditional meta-analysis paradigms, pooling all included studies to calculate a single effect size, concluding that ‘Xiaoyao Formula demonstrates comparable or marginally superior efficacy to SSRIs’. This paradigm implicitly assumes Xiaoyao Formula exerts a homogeneous therapeutic effect across all depression patients—positioning it as a ‘universal antidepressant’. Within this framework, heterogeneity between studies is treated as noise requiring statistical ‘control’ (via random-effects models, subgroup analyses, meta-regression), rather than potential signals carrying clinical information. However, when included studies exhibit systematic differences in formula composition (Xiaoyao Powder, Jiawei Xiaoyao Pills, Dan Zhi Xiaoyao Powder), dosing regimens, control designs (placebo, SSRIs, combination therapy), and patient characteristics (generalised MDD, postpartum depression, perimenopausal depression), the artificially aggregated ‘average effect size’ offers limited guidance for any specific clinical scenario.

This review therefore adopts an alternative analytical approach: abandoning pursuit of a single SMD or RR value in favour of systematically describing the distribution patterns of therapeutic signals across different population subtypes. This strategy reconceptualises inter-study heterogeneity as potential effect-modifying information rather than a statistical interference requiring elimination. Had this review employed conventional methods, it might have reported ‘Xiaoyao Formula SMD = −0.35 [95% CI: −0.52, −0.18], with no statistically significant difference from SSRIs’—a conclusion statistically valid yet entirely obscuring potential differential efficacy signals in the postpartum and perimenopausal subgroups.

Also, the rationale for this strategy is supported by mechanistic investigations: network pharmacology and *in vitro* experiments preliminarily suggest that the PI3K-Akt, NR3C1, and NLRP3 inflammasome pathways may constitute key mechanisms of action for the Xiao Yao formula. The temporal sequence of IL-6 declines preceding symptomatic improvement followed by BDNF elevation aligns with the hypothetical model of ‘inflammation preceding neuroplasticity recovery’. While downregulation of BDNF promoter methylation further suggests epigenetic regulation may participate in mood recovery. This multi-pathway, cascade-synergistic pharmacological profile aligns with the concept of ‘rational polypharmacology,’ concept, suggesting Xiaoyao Formula may simultaneously modulate inflammation, neurotrophic pathways, and the stress axis at lower doses. This may underpin its superior tolerability compared to SSRIs and explain why its therapeutic signals are more pronounced in subtypes with prominent inflammation-endocrine features (postpartum and perimenopausal depression).

### 4.4 Study limitations

The findings of this review should be interpreted with caution in light of several methodological limitations. Although 21 RCTs were included–a relatively substantial number within the field of traditional Chinese medicine antidepressant research–the design characteristics of most trials constrained the strength of evidence: sample sizes were generally small (median approximately 100 cases), making it difficult to detect effect size differences of moderate magnitude or below; intervention periods were concentrated within 6–12 weeks, precluding assessment of long-term maintenance effects and relapse prevention; Over two-thirds employed combination therapy designs (Xiaoyao Formula plus SSRIs versus SSRIs monotherapy), rather than direct monotherapy comparisons of Xiaoyao Formula versus SSRIs, thus obscuring the formula’s independent contribution. Furthermore, the evidence base is almost entirely confined to Han Chinese populations in mainland China. Genetic polymorphisms in drug-metabolising enzymes, variations in gut microbiota composition, and the influence of cultural background on symptom expression and treatment expectations may all limit the generalisability of conclusions to other populations. Given these characteristics, the reported efficacy differences in this review—whether comparable or marginally superior overall remission rates, or potential advantages in specific subtypes—should be interpreted as ‘suggestive’ or ‘indicative’ findings.

Evidence regarding mechanisms similarly faces an inferential gap between association and causation. The sequential pathway proposed herein—‘inflammation downregulation → BDNF epigenetic regulation → PI3K-Akt activation’—is conceptually compelling and theoretically resonates with observed early efficacy and subtype differences. However, supporting data primarily derive from network pharmacology predictions and cross-sectional biomarker association studies. Network pharmacology relies on computational predictions from metabolite-target interaction databases, reflecting theoretical possibilities rather than actual biological events; Although biomarker studies observed IL-6 decline preceding symptom improvement and BDNF methylation status correlating with treatment efficacy, these associations fail to distinguish causal directionality—whether inflammation downregulation constitutes a direct pharmacological effect of Xiaoyao Formula or a secondary physiological adjustment following symptom amelioration remains unresolved by current evidence. Establishing genuine causal chains requires longitudinal multi-time-point sampling within the same cohort, combined with joint modelling of drug exposure concentrations, biomarker dynamics, and symptom trajectories. Evidence of this calibre remains absent in the current literature.

The three-dimensional Q-marker system, based on UPLC-HRMS, represents one of the methodological innovations synthesised in this review. It offers a technical pathway to address the longstanding challenge of batch-to-batch consistency in Chinese botanical drug formulae. However, it is crucial to emphasise that the predictive relationship between chemical metabolite quantification and clinical efficacy is currently considered to be a prospective hypothesis. The hypothesis that attaining designated concentrations of Chaihu saponin A, ferulic acid, and glycyrrhizic acid results in “predictable clinical effects” remains to be substantiated by direct validation. Moreover, no systematic correspondence has been identified between batch-to-batch variability in Q-marker concentrations and the heterogeneity observed in effect size estimation studies. The evolution of the Q-marker system from a quality control tool into a predictive efficacy tool necessitates the establishment of quantitative metabolite-exposure-effect relationships in prospective trials of standardised formulations, a task that is yet to be undertaken.

Furthermore, an integrated precision assessment system combining biomarkers and digital phenotypes remains unestablished, representing another significant gap in current research. Although Yang et al. (2023) proposed that baseline IL-6/BDNF ratios may predict therapeutic response to Xiaoyao Formula, offering preliminary insights for biomarker-guided patient stratification, this metric lacks paired validation with objective behavioural indicators. Discrepancies often exist between patients’ subjective symptom reports and objective functional recovery, rendering single biomarkers inadequate for capturing this complexity. With the rapid advancement of digital health technologies, integrating digital phenotype data—such as sleep-wake rhythms, daytime activity levels, and social interaction frequency monitored via wearables—with blood-based multi-omics biomarkers holds promise for achieving more precise treatment stratification and real-time efficacy monitoring. This dual-track assessment framework of ‘biomarkers + digital phenotypes’ remains in the proof-of-concept stage within antidepressant research, yet may represent a significant future direction for precision medicine studies in traditional Chinese medicine.

### 4.5 Safety profile, adverse effects, and potential interactions

As the reviewer rightly emphasises, the safety and tolerability profile of Xiaoyao-type botanical drug formulations merits dedicated consideration. Across the 21 randomised controlled trials included in the review, the completeness and systematic rigour of adverse event reporting varied substantially. Among the trials that provided structured adverse event data, Xiaoyao-type formulations demonstrated a consistently favourable tolerability profile compared to conventional antidepressant comparators. The most methodologically rigorous head-to-head comparison (Su et al., 2019; n = 210; double-blind, double dummy) reported that Jiawei Xiaoyao capsules were associated with significantly fewer gastrointestinal adverse events (e.g., nausea, diarrhoea and constipation) than sertraline. Notably, no cases of sexual dysfunction were reported, despite this being an adverse effect experienced by approximately 30%–70% of patients treated with SSRIs (Clayton et al., 2002). Fan et al. (2024) reported mild gastrointestinal discomfort in 3.7% of the Xiaoyao Pill group versus 7.4% in the placebo group, with no serious adverse events occurring in either group during the eight-week trial.

Regarding hepatotoxicity, a frequent concern with multi-botanical drug preparations, none of the 21 included trials reported clinically significant elevations in hepatic transaminases (ALT, AST) or other indicators of hepatocellular injury. This finding is consistent with post-marketing pharmacovigilance data from the Chinese National Adverse Drug Reaction Monitoring System, which classifies Xiaoyao Wan as a lower-risk traditional Chinese medicine patent medicine. However, the relatively short intervention durations in the included trials (median 8 weeks, range 4–24 weeks) mean that definitive conclusions about long-term hepatic safety cannot be drawn, and post-marketing surveillance studies with extended follow-up are needed.

Potential pharmacokinetic and pharmacodynamic interactions between Xiaoyao-type formulations and conventional psychotropic medications are a critical safety consideration, particularly since nine of the 21 trials used an add-on design combining the formulations with antidepressants or mood stabilisers. Glycyrrhiza uralensis (gancao) contains glycyrrhizic acid, which has been shown to inhibit 11β-hydroxysteroid dehydrogenase type 2. This can lead to pseudoaldosteronism with hypokalaemia when taken in high doses or for a prolonged period (Stormer et al., 1993). Bupleurum chinense contains saikosaponins that may modulate cytochrome P450 enzyme activity, particularly CYP3A4 (Xin et al., 2021). This could theoretically alter the metabolism of co-administered selective serotonin reuptake inhibitors (SSRIs), benzodiazepines, or carbamazepine. Mentha haplocalyx contains menthol, which is a known inhibitor of CYP2A6 and a weak inhibitor of CYP3A4 (Dresser et al., 2002). No clinically significant drug interactions were reported in the nine add-on trials, including Zhang et al.’s (2007b) combination of FEWP with carbamazepine (n = 235). However, formal pharmacokinetic interaction studies between Xiaoyao-type formulations and conventional psychotropic agents have not been conducted, and the absence of such studies should not be interpreted as evidence that no interactions occur. Future clinical trials should incorporate therapeutic drug monitoring for co-administered psychotropic medications to characterise any potential pharmacokinetic interactions.

While rare, allergic reactions have been reported in post-marketing surveillance for botanical drug preparations containing Angelica sinensis and Bupleurum chinense, primarily manifesting as mild dermatological reactions (urticaria and pruritus). Among the included trials, no anaphylactic or severe allergic reactions were documented. Regarding reproductive safety, Angelica sinensis exhibits oestrogenic activity and uterotonic properties, so Xiaoyao-type formulations should be used with caution during pregnancy—a consideration that is particularly relevant to trial populations with premenstrual syndrome (Li et al., 2024) and infertility (Sun et al., 2012). The overall adverse event profile across the evidence base supports the characterisation of Xiaoyao-type formulations as well tolerated in the short to medium term. However, systematic pharmacovigilance data from adequately powered long-term trials are necessary to establish a comprehensive safety profile suitable for evidence-based clinical recommendations.

### 4.6 Towards precision assessment: biomarkers and digital phenotypes

The three-dimensional Q-marker system, which is based on UPLC-HRMS, is one of the methodological innovations synthesised in this review. It offers a technical pathway that addresses the longstanding challenge of achieving consistent results when producing Chinese botanical drug formulae in different batches. However, it should be noted that the predictive relationship between the quantification of chemical metabolites and clinical efficacy is still a hypothesis. Direct clinical validation of an exposure–response relationship is lacking; it remains unproven that attaining target concentrations of Chaihu saponin A, ferulic acid and glycyrrhizic acid translates into predictable clinical benefit. To date, no evidence has linked batch-to-batch variability in Q-marker concentrations to the between-study heterogeneity in effect-size estimates, largely because concentration data are rarely reported at the level required for quantitative linkage. Transitioning the Q-marker system from a quality control tool to a predictive efficacy tool necessitates the establishment of quantitative metabolite–exposure–effect relationships in prospective trials of standardised formulations.

An integrated precision assessment system combining biomarkers and digital phenotypes remains unestablished, representing another significant gap in current research. Although the biomarker data from Fan et al. (2024) suggest that DNA methylation patterns may predict therapeutic response, these metrics lack paired validation with objective behavioural indicators. Discrepancies—a gap highlighted by Li et al.’s (2008) demonstration that Barthel Index improvement can accompany or precede subjective mood amelioration—between patients’ subjective symptom reports and objective functional recovery render single biomarkers inadequate for capturing the full complexity of treatment response. With the rapid advancement of digital health technologies, integrating digital phenotype data—such as sleep-wake rhythms, daytime activity levels, and social interaction frequency monitored via wearables—with blood-based multi-omics biomarkers holds promise for achieving more precise treatment stratification and real-time efficacy monitoring. This dual-track assessment framework of ‘biomarkers + digital phenotypes’ remain in the proof-of-concept stage within antidepressant research yet may represent a significant future direction for precision medicine studies in traditional Chinese medicine.

### 4.7 Future research directions

Existing RCTs predominantly span 8–12 weeks, with dose-response relationships and maintenance therapy value yet to be systematically evaluated; insufficient follow-up also constrains assessments of relapse prevention efficacy. Samples primarily derive from Han Chinese populations, lacking cross-ethnic external validation.

Subsequent studies should employ stratified randomised trials combining IL-6/BDNF ratios with digital phenotype metrics, utilising multi-dose stepwise designs with ≥24 weeks’ follow-up, and validate generalisability across multinational centres. Regarding quality control, the predictive efficacy of the established three-dimensional Q-marker system for treatment response fluctuations requires confirmation through prospective consistency trials.

## 5 Conclusion

This systematic review, based on 21 randomised controlled trials and three mechanism studies, provides a comprehensive assessment of the clinical evidence for Xiao Yao-type formulas in treating depressive disorders. It attempts to integrate efficacy data with mechanism research to generate testable working hypotheses. The synthesised evidence indicates that Xiaoyao-type formulas demonstrate comparable overall remission rates to SSRIs, or show a slight advantage in certain combined designs. They consistently exhibit superior tolerability—particularly regarding gastrointestinal adverse reactions and sleep disturbances. The trend towards earlier onset of efficacy reported in some trials (1–2 weeks versus 2–4 weeks) holds significant clinical potential should it be confirmed in future specifically designed studies.

However, this review’s core contribution lies not in the overall efficacy estimates, but in proposing an integrated framework repositioning the clinical value of Xiaoyao San. Subtype analysis reveals that the efficacy signals of Xiaoyao formulas are more pronounced in postpartum depression and perimenopausal depression—two subtypes characterised by prominent inflammation-endocrine dual dysregulation—precisely the domains where traditional SSRIs exhibit relatively limited efficacy. Mechanistic studies suggest a multi-target pharmacological profile—downregulation of IL-6 and NLRP3, epigenetic regulation of BDNF, and activation of the PI3K-Akt pathway—theoretically consistent with the complex pathophysiology of these subtypes. Based on these observations, this review proposes that Xiaoyao San’s clinical value may not lie in being a ‘superior antidepressant,’ but rather in its enhanced alignment with a pathophysiological dimension relatively neglected by current treatments—inflammation-endocrine dysregulation. Perimenopausal and postpartum depression may constitute its preferred indications.

This hypothesis remains at the conceptual stage, its validity clouded by multiple limitations, including the strength of the evidence, mechanistic inference and quality control. The included studies generally featured small sample sizes, short intervention periods, predominantly combined medication designs and exclusivity to Han Chinese populations. The sequential pathway hypothesis primarily relies on network pharmacology predictions and association studies, lacking established causal chains. Future research should prioritise the following directions: Conduct prospective validation trials in predefined inflammatory-endocrine subtype populations, using time to onset as the primary endpoint; Incorporating longitudinal multi-time point measurements of inflammatory markers (IL-6, CRP) and neurotrophic factors (BDNF) to validate the sequential pathway hypothesis; Establishing quantitative relationships between Q-marker concentrations and clinical effects; Exploring precision assessment frameworks integrating biomarkers with digital phenotypes. Should the ‘inflammation-endocrine subtype hypothesis’ proposed in this review be supported by these studies, it would not only provide more precise patient selection criteria for the evidence-based application of Xiaoyao San, but also potentially establish a new methodological paradigm for evaluating multi-metabolite natural medicines—shifting the focus from questioning ‘whether it is effective’ to answering ‘for whom it is effective’.

## Data Availability

The original contributions presented in the study are included in the article/Supplementary Material, further inquiries can be directed to the corresponding author.
