# Supplementary material for: Inflammation–neurotrophin synergy of Xiao-yao-san-type botanical drug formulations in depressive disorders: a qualitative synthesis of recent human studies with taxonomic and compositional characterisation
Source: Front Pharmacol. 2026 Mar 18;17:1718573. doi: 10.3389/fphar.2026.1718573 (PMC13038987; doi:10.3389/fphar.2026.1718573)
Supplement: Supplementary file 1 [file Table1.pdf]

Supplementary Table 1. Detailed Characteristics of All 21 Included Randomised Controlled Trials

Study (Author, Year)	Clinical Population	n	Intervention (Formulation)	Comparator	Study Design	Duration (weeks)	Primary Outcome Measures	Key Findings	Overall RoB
Placebo-Controlled Monotherapy Trials (k = 8)									
Fan (2024)	MDD (mild–moderate; DSM-5 criteria)	108	Xiaoyao Pills (standard patent medicine)	Matched placebo capsules	Double-blind RCT	8	HAMD-17; genome-wide DNA methylation (Illumina 450K array)	Significant HAMD-17 improvement vs placebo; DNMT1 upregulation and genome-wide methylation normalisation in peripheral blood leukocytes; first epigenetic evidence for Xiaoyao formulas	**Low**
Li Y. (2022)	Mixed anxiety-depressive disorder	400	Shugan granule (Xiaoyao-derived preparation)	Matched placebo granules	Double-blind, multicentre RCT	8	HAMD; HAMA	Significant reduction in both anxiety and depression scores vs placebo; largest Xiaoyao-family trial to date (n = 400)	**Low**
Du (2014)	Perimenopausal functional dyspepsia with comorbid depression	180	Xiaoyao Pill	Matched placebo	Double-blind RCT	8	HRSD; gastric motility parameters (motilin, gastrin, gastric emptying rate)	Concurrent improvement in depression scores and gastric motility parameters vs placebo; dual-endpoint design supporting somatic–affective co-regulation	**Low**
Li X.Y. (2024)	Premenstrual syndrome (TCM pattern: liver depression, spleen deficiency, blood-heat)	144	Jiawei Xiaoyao Pill	Matched placebo	Double-blind, multicentre RCT	3 cycles	Daily Record of Severity of Problems (DRSP)	Significant PMS symptom improvement vs placebo across three menstrual cycles	**Low**
Yang (2023)	Post-COVID-19 recovery with sleep and mood disturbance	200	Xiaoyao capsule	Matched placebo	RCT	4	Sleep quality scales; TCM syndrome differentiation scales	Improved sleep disorders and mood disturbance in post-COVID patients vs placebo	**SC**
Chen G. (2020)	Functional dyspepsia with comorbid mood symptoms	144	Jiawei Xiaoyao botanical drug formulation	Matched placebo	Double-blind, multicentre RCT	4	FD symptom scales; mood symptom measures	Significant improvement in functional dyspepsia symptoms and	**Low**

								comorbid mood disturbance vs placebo	
Zhang Z.-J. (2007b)	Mood disorders (unipolar and bipolar depression)	149	Free and Easy Wanderer Plus (FEWP / Jiawei Xiaoyao San)	Matched placebo capsules	Double-blind RCT	12	HAMD; MADRS; CGI-S	74% vs 42% response rate (FEWP vs placebo); significant improvement on all three validated outcome scales over 12 weeks	SC
Chen J.-X. (2005)	Liver-Stagnation-Spleen-Deficiency Syndrome (LSSDS; TCM diagnosis)	58	Chai hu (Radix Bupleuri) containing formulation	None (single-arm biomarker study)	Single-arm biomarker study	NR	Plasma beta-endorphin, epinephrine, dopamine	Multi-neurotransmitter modulation: altered plasma beta-endorphin and dopamine concentrations following treatment	SC
Head-to-Head Comparison Trials (k = 4)									
Su (2019)	MDD (DSM-IV criteria)	210	Jiawei Xiaoyao capsules	Sertraline	Double-blind, double-dummy, multicentre RCT	8	HAMD-17	Non-inferior to sertraline; comparable remission rates with fewer reported adverse events (particularly GI and sleep disturbance)	Low
Luo (2006)	Major depressive disorder	63	Danzhi Xiaoyao San	Maprotiline	Head-to-head comparison	6	HAMD	Comparable antidepressant efficacy to maprotiline with better tolerability profile	SC
Li L.-T. (2008)	Post-stroke depression	150	Free and Easy Wanderer Plus (FEWP)	Fluoxetine; Placebo (3-arm)	Double-blind, 3-arm RCT	8	Hamilton Depression Scale (HDS); Barthel Index	Both FEWP and fluoxetine superior to placebo; FEWP showed faster symptom onset at week 2; concurrent improvement in functional independence (Barthel Index)	SC
Li Y.-J. (2007)	MDD (companion biomarker study to Luo 2006)	63	Danzhi Xiaoyao San	Maprotiline	Head-to-head with biomarker assessment	6	HAMD; IL-2, IL-6, cortisol, ACTH, T-lymphocyte subsets	Multi-system neuro-immuno-endocrine modulation: altered IL-6, cortisol, ACTH, and T-cell subsets concurrent with mood improvement	SC

Add-on / Augmentation Trials (k = 9)									
Zhang Z.-J. (2007a)	Bipolar disorders (including 124 with bipolar depression)	235	FEWP + carbamazepine	Carbamazepine + matched placebo	Double-blind, multicentre RCT	12	HAMD; MADRS; YMRS; CGI-S	84.8% vs 63.8% augmentation response (FEWP+CBZ vs PBO+CBZ); significant improvement on depression scales without mania induction	SC
Yi (2010)	Major depressive disorder	190	Chaihu Xiaoyao mixture + paroxetine	Paroxetine alone	Open-label	8	HAMD	Enhanced antidepressant response with combination therapy vs paroxetine monotherapy	SC
Zhang (2006)	Senile depression (plateau district population)	90	TCM botanical drug formula (Xiaoyao-type) + fluoxetine	Fluoxetine alone	Open-label	8	HAMD	Added benefit of TCM augmentation over fluoxetine monotherapy in elderly patients at high altitude	SC
Yang (2007)	Depression (unspecified diagnostic criteria)	64	Modified Xiaoyao Pill + amitriptyline	Amitriptyline alone	Open-label	6	HAMD; treatment compliance assessment	Improved treatment compliance and enhanced antidepressant efficacy with combination therapy vs amitriptyline alone	SC
Yu (2007)	Depression (unspecified diagnostic criteria)	105	Modified Xiaoyao Decoction + clomipramine	Clomipramine alone	Open-label	8	HAMD	Enhanced response rate and improved tolerability with combination therapy vs clomipramine monotherapy	SC
Liu Y.-L. (2008)	Hypertension with comorbid depression	NR	Danzhi Xiaoyao San + antihypertensive medication	Antihypertensive alone	Open-label	NR	HAMD; blood pressure measures	Concurrent improvement in depressive symptoms and blood pressure control with combined treatment	SC
Sun (2012)	Infertility-related psychological distress (IVF patients)	97	Acupuncture + Xiaoyao-type TCM + IVF protocol	IVF protocol alone	Open-label	NR	Anxiety and depression scales; IVF treatment outcomes	Reduced psychological distress during IVF cycle; trend toward improved treatment outcomes	SC
Gao (2024)	Burning mouth syndrome with comorbid anxiety/depression	78	Danzhixiaoyao tablets + methylcobalamin	Methylcobalamin alone	Open-label RCT	NR	BAI; BDI; BMS symptom scales	Improved BMS symptoms and comorbid anxiety/depression scores vs	SC

								methylcobalamin alone	
Liu W.-D. (2019)	Oral lichen planus with comorbid anxiety/depression	60	Modified Xiaoyao Pill + standard oral treatment	Standard oral treatment alone	Open-label	NR	OLP clinical assessment scales; anxiety and depression scales	Improved OLP lesion scores and comorbid mood symptoms vs standard treatment alone	**SC**

Note: All 21 studies were conducted in China. SC = some concerns; RoB = risk of bias; DB = double-blind; DD = double-dummy; MC = multicentre; RCT = randomised controlled trial; FEWP = Free and Easy Wanderer Plus (Jiawei Xiaoyao San); FD = functional dyspepsia; PMS = premenstrual syndrome; BMS = burning mouth syndrome; OLP = oral lichen planus; LSSDS = Liver-Stagnation-Spleen-Deficiency Syndrome; IVF = in vitro fertilisation; HAMD = Hamilton Depression Rating Scale; HAMA = Hamilton Anxiety Rating Scale; MADRS = Montgomery–Åsberg Depression Rating Scale; CGI-S = Clinical Global Impression–Severity; BAI = Beck Anxiety Inventory; BDI = Beck Depression Inventory; HDS = Hamilton Depression Scale; HRSD = Hamilton Rating Scale for Depression; DRSP = Daily Record of Severity of Problems; YMRS = Young Mania Rating Scale; CBZ = carbamazepine; NR = not reported. Risk of bias assessed using the Cochrane Risk of Bias 2.0 tool (Sterne et al., 2019). Six studies (28.6%) achieved overall low risk; 15 (71.4%) raised some concerns; none was rated high risk.
